# Evaluating potential of leaf reflectance spectra to monitor plant genetic variation

**DOI:** 10.1186/s13007-023-01089-9

**Published:** 2023-10-14

**Authors:** Cheng Li, Ewa A. Czyż, Rayko Halitschke, Ian T. Baldwin, Michael E. Schaepman, Meredith C. Schuman

**Affiliations:** 1 Department of Geography, Faculty of Science, University of Zurich, Winterthurerstrasse 190, 8057 Zurich, Switzerland; 2 Department of Molecular Ecology, Max Planck Institute for Chemical Ecology, Hans-Knoell-Strasse 8, 07745 Jena, Germany; 3 Department of Chemistry, Faculty of Science, University of Zurich, Winterthurerstrasse 190, 8057 Zurich, Switzerland

**Keywords:** Leaf reflectance, Field spectroscopy, Genetic variation, Genotype x environment interactions, Multiparent Advanced Generation Inter-Cross (MAGIC), Transgenic plants, *Nicotiana attenuata*

## Abstract

Remote sensing of vegetation by spectroscopy is increasingly used to characterize trait distributions in plant communities. How leaves interact with electromagnetic radiation is determined by their structure and contents of pigments, water, and abundant dry matter constituents like lignins, phenolics, and proteins. High-resolution (“hyperspectral”) spectroscopy can characterize trait variation at finer scales, and may help to reveal underlying genetic variation—information important for assessing the potential of populations to adapt to global change. Here, we use a set of 360 inbred genotypes of the wild coyote tobacco *Nicotiana attenuata*: wild accessions, recombinant inbred lines (RILs), and transgenic lines (TLs) with targeted changes to gene expression, to dissect genetic versus non-genetic influences on variation in leaf spectra across three experiments. We calculated leaf reflectance from hand-held field spectroradiometer measurements covering visible to short-wave infrared wavelengths of electromagnetic radiation (400–2500 nm) using a standard radiation source and backgrounds, resulting in a small and quantifiable measurement uncertainty. Plants were grown in more controlled (glasshouse) or more natural (field) environments, and leaves were measured both on- and off-plant with the measurement set-up thus also in more to less controlled environmental conditions. Entire spectra varied across genotypes and environments. We found that the greatest variance in leaf reflectance was explained by between-experiment and non-genetic between-sample differences, with subtler and more specific variation distinguishing groups of genotypes. The visible spectral region was most variable, distinguishing experimental settings as well as groups of genotypes within experiments, whereas parts of the short-wave infrared may vary more specifically with genotype. Overall, more genetically variable plant populations also showed more varied leaf spectra. We highlight key considerations for the application of field spectroscopy to assess genetic variation in plant populations.

**Supplementary Information:**

The online version contains supplementary material available at 10.1186/s13007-023-01089-9.

## Introduction

**Fig. 1:**
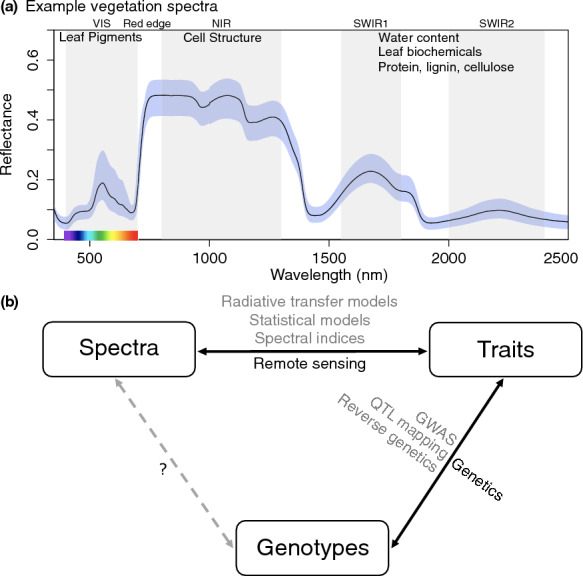
Framework for this study. **a** Example vegetation reflectance spectra (line = mean, blue shaded region = range for biological replicates) across wavelengths from 350 nm to 2500 nm, indicating biochemical and structural influences on different features within different optical ranges (shaded grey: visible = VIS, near infrared = NIR, short-wave infrared = SWIR). The red edge is located at the boundary between VIS and NIR, typically falling within the 700–730 nm range for vegetation. **b** We are interested in developing approaches alongside existing tools in remote sensing and genetics, towards linking plant spectra to genotypes

As synthesized by Jaquemond and Ustin [1], great natural variation in the color, texture, and form of leaves corresponds to phylogenetic differences, reveals symptoms of plants under stress from nutrient or water deficiency or from herbivores and pathogens, and is characterized by phenological shifts like the unfurling of leaf buds—green, white, brown, or black - into green leaves, or the colors of leaf senescence at the end of a growing season. These optical properties, as well as many which are outside the range of visible light, result from features of leaf biochemical composition and structure. The optical domain of solar radiation ranging from 350 nm to 2500 nm comprises three parts past the edge of UVA radiation (320–400 nm). The visible range (VIS, 400–700 nm) is characterized by strong absorption of light by pigments. In the near infrared “plateau” (NIR, 800–1300 nm) absorption is limited to dry matter and water but the multiple scattering within the leaf, related to the fraction of air spaces, i.e., to the internal structure, drives reflectance and transmittance of light. The middle or short-wave infrared (SWIR) is also a zone of strong absorption, primarily by water in a fresh leaf, overlaid with the absorption by dry matter which becomes more apparent in a dried leaf (Fig. 1). The SWIR1 (1550–1800 nm) and SWIR2 (2000–2400 nm) regions are separated by major water absorption bands [2]. Leaf dry matter content refers to a variety of organic compounds including phenolics, cellulose, and lignin, and nitrogen-containing compounds including proteins; generally, any component which comprises ca. 1% or more of dry mass may have a measurable contribution [3]. Leaf biochemical composition and structural components which influence leaf optical properties are well studied and are known to be controlled by developmental, metabolic, and signaling gene variants and their regulation by environmental factors [4]. Related indices of vegetation characteristics have an even longer history of use in earth observation, such as the commonly used normalized difference vegetation index (NDVI) [5], which is widely used in remote sensing to assess and monitor plant growth, vegetation cover, and biomass production from satellite imagery, providing critical information for environmental management and study [6].

Recently, traits observable via the optical properties of plants, often coupled with light detection and ranging (LiDAR) for high-resolution structural information, have been used to survey plant community structural and biochemical composition and to assess functional or species diversity from aerial images [7]. Spectroscopy has shown potential for assessing genetic variation, with studies demonstrating its use in identifying Arabidopsis mutants having altered leaf pigment status [8], differentiating populations within the oak genus (*Quercus*) [9], studying heritable variation in Scots pine (*Pinus sylvestris* L.) [10], and finding correlations between airborne imaging spectroscopy and microstatellite-derived genotype groups [11] or genetic distances [12]. A comprehensive study by Meireles and colleagues integrated leaf spectra with phylogeny on a global scale, revealing evolutionary dynamics of leaf chemistry and structure that can be detected using spectroscopy [13]. All of these studies indicate that leaf spectra have the potential to reveal genetic variation among plants. Furthermore, imaging spectroscopy used in remote sensing has the potential to cover large areas within short times and thus offers unique possibilities for timely and repeated monitoring of biodiversity under global change. However, it is challenging to distinguish environmental and genetic influences on variation in plant and leaf spectra, and the detection of spectra by aerial or spaceborne remote sensing adds additional layers of uncertainty [12]. Thus, controlled studies determining genetic contributions to variation in leaf spectra are needed. Figure 1b shows the conceptual framework for this study.

Here, we employed a subset of the genetic tools developed over decades by Ian Baldwin’s group for the ecological model plant *Nicotiana attenuata* Torr. ex. Wats., the wild coyote tobacco. We used leaf spectroscopy to phenotype a multi-parent advanced generation inter-cross (MAGIC) genetic mapping population and its parental lines, which capture a wide range of phenotypic variation within this species [14]. We also measured leaf spectra from several *N. attenuata* transgenic lines knocked down in genes whose products should affect leaf optical properties, and which have been previously characterized in both glasshouse and field conditions (Table 1). Our experiments were conducted in both more controlled (glasshouse) and field conditions. In the context of comparing results from transgenic lines to results from natural genetic variants, and because we aimed to study plants in a natural setting, we avoided extreme manipulations which would endanger plant viability in the field. For example, genes involved in chlorophyll and carotenoid biosynthesis were described in the 1990’s [15] and knocking down PHYTOENE DESATURASE (PDS) is known to produce white plants, presumably due to photobleaching, even under low light [16]. However, plants with pronounced deficiencies in chlorophyll or carotenoids are unlikely to be viable in many natural environments. In particular, *N. attenuata* inhabits high-light, nutrient-rich, post-fire semi-arid deserts. In the following, we introduce genes which were manipulated in this study.

The protein RuBPCase activase (RCA) controls the activity and turnover of the carbon dioxide-fixing photosynthetic protein RuBisCO in plants [17]. RCA thereby controls both photosynthetic activity as well as leaf protein content, because RuBisCO is the most abundant protein in leaves, and so can be expected to impact both the VIS and NIR, and especially the SWIR regions of the spectrum.

Lignin contributes ca. 1–10% to leaf dry matter content and is important to the structural integrity of leaves and stems [18–20]. Lignins comprise long chains of phenolic building blocks and specific phenolic monomers may be flexibly incorporated [20–23]. Lignins and other phenolic and polyphenolic compounds which act as defense compounds, antioxidants, and sunscreens in leaves and on their surface also influence leaf optical properties in the SWIR2 region; for example, leaf lignin content has been related to absorption features at 1120, 1200, 1420, 1450, 1690, 1940, and 2100 nm ([1, 24] Table 3.4). The protein CINNAMYL ALCOHOL DEHYDROGENASE (CAD) catalyzes a key step in canonical lignin biosynthesis. When CAD is silenced in *N. attenuata*, lignin composition changes, resulting in plants unable to stand upright in the absence of UV-B radiation, and which stand upright but form red stems with exposure to UV-B due to the stimulation of lignin biosynthesis from atypical phenolic monomers [20].

The proteins CHALCONE SYNTHASE (CHAL) and HYDROXYCINNAMOYL-CoA QUINATE TRANSFERASE (HQT) catalyze rate-limiting steps in the synthesis of phenylpropanoids and flavonoids generally (CHAL), and chlorogenic acid specifically (HQT) [25–27]. *N. attenuata* plants modified to knock down their expression of CHAL (irCHAL) do not increase production of phenolic compounds in response to UV-B and may be more susceptible to tissue damage and growth inhibition by UV-B [28, 29]. Thus knocking down expression of CAD, CHAL, or HQT is expected to alter leaf reflectance in the NIR region dominated by leaf structural features, as well as in the SWIR region which is influenced by leaf dry matter content.

Phytohormone signaling modulates regulatory and biosynthetic protein activities in response to developmental and environmental stimuli. In particular, the jasmonate hormones mediate the reduction of photosynthesis and general metabolism and induction of specialized metabolism in response to many biotic stressors such as herbivore attack, in addition to their roles in floral development and fertility [30]. Jasmonates also play developmental roles related to defense such as regulating the density of leaf glandular trichomes, and countering the growth-promoting action of auxins [31]. Jasmonates are produced via an oxylipin biosynthesis pathway from precursor fatty acids cleaved from plastid membranes, and the proteins ALLENE OXIDE CYCLASE (AOC) and a specific lipoxygenase (in *N. attenuata*, LIPOXYGENASE 3 or LOX3) perform rate-limiting steps in jasmonate biosynthesis [31, 32]. The action of jasmonates is modified by the gaseous hormone ethylene, which is produced from the precursor 1-aminocyclopropane-1-carboxylic acid (ACC) by ACC OXIDASE (ACO) [33]. In addition to modulating herbivory-induced wound healing and defense responses, ethylene also regulates the production of phenolic compounds interactively with jasmonates [34–36]. Using genetic modification to abrogate expression of genes involved in jasmonate or ethylene biosynthesis could have wide-ranging contextual effects on leaf reflectance. When investigating leaves of a standardized developmental stage without recent damage from herbivores, we would expect the effects of knocking down ethylene biosynthesis (ACO) may have pronounced effects on the basal accumulation of phenolic compounds affecting the SWIR, while knocking down jasmonate signaling (AOC, LOX3) is expected to have broad effects across the entire VIS-SWIR spectrum.

In this study, we ask how well we can explain variation in leaf spectra by plant genetic variation, using both natural genetic variants as well as transgenic variants targeting specific gene expression in otherwise-isogenic lines, both across and within several different sets of experimental conditions. Specifically, we used 360 inbred genotypes of the ecological model plant *N. attenuata* generated via two different approaches: a forward genetics approach to establish a MAGIC genetic mapping population comprising recombinant inbred lines (RILs) derived from natural genetic variants (parental lines, PLs); and a reverse genetics approach to generate transgenic lines (TLs) with targeted changes in gene expression in otherwise-isogenic plants. To be able to make comparisons among different experiments, we grew an inbred line, used to generate all transgenics and included as a PL, in all three environments as a reference (Utah wild-type, UT-WT). Furthermore, we used two different empty vector (EV) lines as a reference for the TLs. In an initial experiment, the PLs and TLs were measured in a standardized glasshouse environment with lighting to mimic natural sunlight including UV-B, and the differences in within-group patterns and variance in leaf reflectance were compared to the reference genotypes for groups of plants varying substantially in their genomes (PLs) or else differing primarily in their transcriptomes (TLs). TLs, PLs and RILs were then measured in two independent field experiments in different plantations within the plant’s native habitat: one established for the field release of transgenic plants (southwestern Utah) and the other for experiments with the RIL populations (central Arizona). Seven TLs were measured alongside co-planted and size-matched EV reference plants in replicate, repeatedly over multiple days, in order to capture variance over time in the same plants, which might be important to detect differences caused primarily by changes to gene expression patterns. Different standardized (older or younger) leaf positions were also measured in a set of UT-WT plants grown in the same environment, to assess the effect of leaf choice on reflectance spectra. In Arizona, two sibling RIL populations, each comprising 325 RILs, were measured alongside the PLs and additional, co-planted and distributed replicates of the UT-WT using a high-throughput screening approach with freshly harvested, hydrated, cut leaves. This experiment was used to describe patterns of spectral variance for populations with variable (PLs, RILs) versus invariant (UT-WT) genomes, and to compare spectral variation in the parental (PL) and offspring (RIL) populations. We used Principal Component Analysis (PCA) on all three datasets and Analysis of Variance (ANOVA) across UT-WT samples to investigate variance across datasets and the contribution of experimental conditions. Within environments, we used Coefficient of Variation (CV) and measurement uncertainty analysis as well as PCA to characterize variance within experiments, followed by linear models to assess the effects of genetic variation in comparison to other within-experiment effects. The effects we investigated, including measurement time and leaf choice , were determined based on the specific design and aims of each experiment.

## Material and methods

### Plant material

We measured inbred lines of *Nicotiana attenuata* Torr. ex S. Watson generated using two different approaches by the Molecular Ecology department at the Max Planck Institute for Chemical Ecology, Jena. A forward genetics approach established a 26-parent Multiparent Advanced Generation Intercross (MAGIC) population comprising 325 recombinant inbred lines (RILs) capturing natural variation within the species [14]. A reverse genetics approach used *Agrobacterium*-mediated transformation followed by selfing to generate homozygous transgenic lines (TLs) to study the consequences of changes in the expression of specific genes [37].

#### Multiparent Advanced Generation Intercross (MAGIC)

In MAGIC designs, multiple inbred founders are intercrossed several times in a specified order to combine genetic material of all the founders in each single descendent line [38]. This leads to highly diverse genotypes each with a unique mosaic of founder alleles. In this study, the MAGIC population captures the majority of the phenotypic diversity measured among approximately 400 natural accessions of *N. attenuata* that have been collected over the past three decades of fieldwork, as described by Ray and colleagues [14]. Diallelic crossing was performed on 26 PLs (each PL crossed with every other), resulting in a population of 325 RILs (26 × 25/2), each harboring genetic contributions from all 26 PLs in their genome. Two of six sibling populations were selected and inbred for six subsequent generations to ensure mean 99% homozygosity across all loci. For comparison, a 30x inbred line was used, derived from seeds originally collected from natural populations of *N. attenuata* from the Desert Inn Ranch near Santa Clara, UT, USA [39]. This line, here called UT-WT (short for Utah wild-type), has been used as the reference wild-type line for decades in the *N. attenuata* system.

#### Transgenic lines (TLs)

The TLs used in this study were derived from inbred generations of the UT-WT line described above. Gene expression was knocked down using inverted repeat (ir) RNA interference (RNAi) constructs, and two lines each containing an insertion of the vector without an RNAi construct (empty vector, EV) were used as controls, as described (Table 1 and references therein). We selected lines with targeted alterations in physiological processes expected to influence leaf optical properties via biosynthesis of polyphenols (irHQT), phenylpropanoids and flavonoids (irCHAL), and lignin (irCAD); regulation of the photosynthetic protein RuBisCO (irRCA); or the regulation of these and other environmentally responsive processes via hormonal signaling pathways (irACO, irLOX3, irAOC) (Table 1).

**Table 1:** Transgenic lines used in this study

Genotype	Gene	Process	Line and reference
irACO	ACC oxidase 1, NIATv7_g18038.t1	Ethylene biosynthesis	A-03-321-10-1, [33]
irHQT	Hydroxycinnamoyl-CoA quinate transferase, NIATv7_g12918.t1	Chlorogenic acid biosynthesis	A-13-153-6-3, [27]
irCHAL	Chalcone synthase, NIATv7_g39367.t1	Phenylpropanoid and flavonoid biosynthesis	A-06-283-1-2, [25]
irLOX3	Lipoxygenase 3, NIATv7_g32174.t1	Oxylipin biosynthesis (OPDA and jasmonates)	A-03-562-2, [40]
irAOC	Allene oxide cyclase, NIATv7_g34489.t1	Oxylipin biosynthesis (OPDA and jasmonates)	A-07-457-1-2, [41]
irCAD	Cinnamyl alcohol dehydrogenase, NIATv7_g23398.t1	Lignin biosynthesis	A-07-206-1, [20]
irRCA	RuBisCo activase, NIATv7_g06559.t1	Photosynthesis (CO$^{2}$ fixation)	A-03-462-7-1, [17]
pRESC2NC	Empty vector control	Transformation control	A-03-009-1-2, [42]
pSOL3NC	Empty vector control	Transformation control	A-04-266-3-5, [42]

#### Reference genotypes

**Fig. 2:**
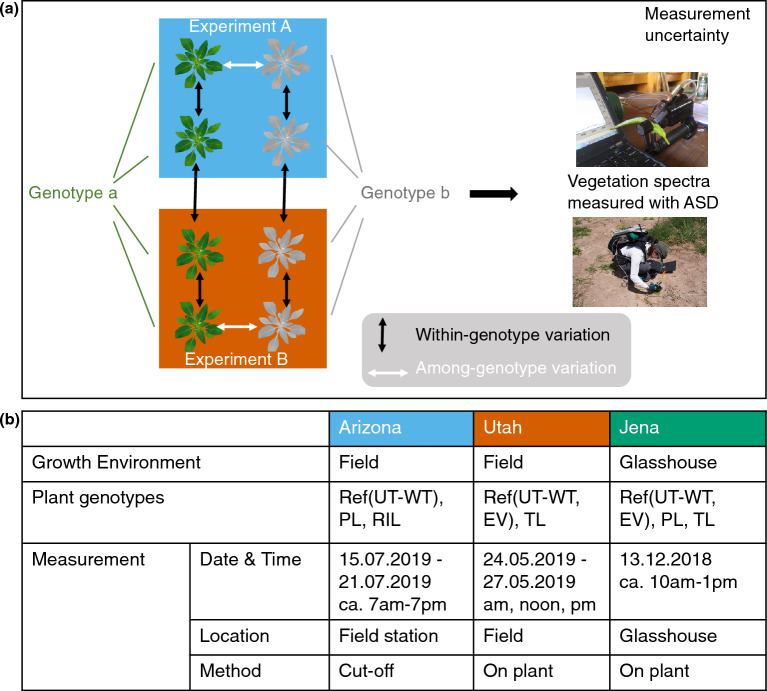
Overview of datasets. **a** Conceptual overview of the datasets, which allows for comparison between genotypes within experimental environments, and within genotypes among experimental environments, incorporating estimated measurement uncertainty. ASD, an abbreviation for Analytical Spectral Devices. **b** Summary of dataset characteristics

To compare spectral variation within and between experimental conditions, we grew UT-WT plants in all three environments (for details, see Fig. 2). Two different EV lines, which were derived by inserting two different empty transformation vectors into the UT-WT, and the 30x inbred UT-WT were included in the Glasshouse experiment in Jena. One of the EV lines and the 30x inbred UT-WT were used in Field_UT, and the 30x inbred UT-WT was used in Field_AZ as reference. We refer to the 30x inbred UT-WT (from here on, simply UT-WT) and the two EV lines as the reference genotypes, or Ref for short. These “references” were replicated and measured interspersed with the other plants measured during field and glasshouse experiments. Differences measured within the reference population thus indicate variation within experimental environments and within genotypes over the course of our measurements, including technical variation.

### Growth conditions and environments

The plants were grown in three different environments: a glasshouse in Jena, Germany, a field plantation near Prescott, Arizona, USA, or a field plantation near St. George, Utah, USA. The three environments are referred as Glasshouse, Field_AZ, and Field_UT. The data generated from the three environments are referred as the Glasshouse dataset, Field_AZ dataset, and Field_UT dataset. The whole dataset refers to these three datasets combined. For analyses of diurnal effects, measurements from 7 am to 12 pm counted as “am”, from 12 pm to 4 pm as “noon” (afternoon), and from 4 pm to 8 pm as “pm” (late afternoon/evening).

#### Jena glasshouse

The glasshouse was located at the Max Planck Institute for Chemical Ecology, Jena, Germany (50.911260, 11.569520). Measurements in the glasshouse comprised an initial screening of lines and populations for use in this study and were conducted on one to three plants per genotype (three per genotype for all transgenic lines and UT-WT, one per genotype for all parental lines of the MAGIC population) in December 2018 prior to field experiments in 2019. RILs of the MAGIC population were in the final phase of inbreeding at the time of these measurements, prior to seed collection for field plantations, and were not included in the glasshouse experiment. Seeds were germinated directly in potting soil (8 × 13 Teku flats) imbibed from below with a solution from native soil collected in the Utah field environment, prepared by stirring 10 mL of soil stored in the dark at room temperature in 1 L of tapwater for 1 h, allowing to settle for 30 mins and then decanting the supernatant into a new bottle; the supernatant was used to imbibe the soil for ca. 2 h (1 L per flat). Seeds were incubated in a germination cue solution of sterilized diluted liquid smoke (House of Herbs, diluted 1:50), 5 mL for each seed lineage in a 15 mL culture tube, with GA3 (10 $\mu$L/mL of a 0.1 M solution in ethanol) for 1 h; this solution was decanted and 5 mL per culture tube of fresh sterilized 1:50 diluted liquid smoke was added and seeds were left in the smoke solution overnight. Seeds were sown directly from the smoke solution onto imbibed soil in Teku pots by placing gently onto the top of the soil with a glass Pasteur pipette, one seed per Teku pot. Line positions were indicated with plastic labels in pots as well as with a layout map which was kept together with the pots. Germination proceeded and seedlings grew large enough for transfer to 1 L pots over a period of 18 d. During this period, trays containing Teku pots were kept in the glasshouse on a common germination table at 23–25$^{\circ }$C daytime/19–23$^{\circ }$C nighttime temperatures under 16 h supplemental lighting from Master Sun-T PIA Agro499 or Plus 600W Na light (Philips, Turnhaut, Belgium). Clear plastic lids were kept closed over Teku trays for the first 5 d, then opened gradually over 2 d and finally removed by glasshouse staff who also monitored the Teku pots for sufficient moisture by watering from below as needed. From the second week a dilute fertilizer was used for watering: 3 g/10 L Peter’s Allrounder 20−20−20+TE (www.scotsprofessional.com).

Seedlings were transferred to potting soil in 1 L pots on the 18th day and moved to a table underneath LED supplemental lighting (Valoya NS-1 LEDs) to maintain a spectrum similar to the outside solar spectrum, and under the same temperature and photoperiod as for germination. Water with fertilizer was provided from below by a drip irrigation system. Fertilization of potted plants was previously described [37]. Plants were additionally exposed to UV-B light at a low dose so as to stimulate the production of UV-responsive compounds without damaging vegetation, as the glasshouse panels absorb UV-B light. UV-B treatment was done for two days at the end of the rosette stage of growth and beginning of bolting (32 and 33 d after sowing, on December 11th and December 12th, 2018) using the same system as Santhanam and colleagues [28] but for two one-hour periods close to solar noon (10:00–11:00, 12:00–13:00) on each day, and plants were measured the day following the second UV-B exposure.

#### Arizona field plantation

The Arizona field site was located at the Walnut Creek Center for Education and research (WCCER) near Prescott, Arizona, USA, within the native range of *N. attenuata* [14], managed in cooperation with the Southwest Experimental Garden Array (SEGA). Germination was conducted in peat pots (Jiffy-7®, http://www.jiffypot.com/) in the week of April 19th, 2019 and otherwise identically as described for the glasshouse in Jena, with the modification that Jiffy pots were kept in closed transparent plastic boxes (Sterilite 32 Quart Latch) first in shade, and then moved to sunlight once true leaves were visible, at which point the lids were removed from the plastic boxes during sunlit hours of the day; temperature was maintained in the boxes by floating them in shallow pools of water. Hardened-off seedlings ca. 1.5–2 cm in diameter were planted on May 14th–17th 2019 into holes in a black weed-suppressing cloth (DeWitt 12-year weed barrier, 4′ x 300′ [12 YR-4300]) covering the field plot. Details of growth conditions were previously described by He and colleagues [43] and Bai and colleagues [44]. The plantation comprised two replicates each of two sibling MAGIC RIL populations, M1 and M2, as well as the PLs used to generate these RILs; and plants of the inbred UT-WT line as reference samples (Ref). Plants were planted in 4-plant clusters (Additional file 1: Fig. S1a), each watered by one dripper from a drip line, consisting of three of the RIL and PL plants and one UT-WT plant in a random configuration [44].

#### Utah field plantation

The Max Planck field station in southwest Utah, USA is situated within the native range of *N. attenuata* [14], at the Lytle Ranch Preserve (plot corners: 37.145504, $-$114.021260; 37.145305, $-$114.020760; 37.144964, $-$114.021602; 37.144800, $-$114.021077) owned and operated by Brigham Young University, Provo, Utah. Germination was conducted as in Arizona; seedling cultivation, hardening-off and planting were conducted similarly and hardening-off and planting procedures are described in more detail by McGale and colleagues [45]. Germination was conducted on March 16th, 2019. The plantation comprised replicates of seven transgenic lines, including an EV line (pRESC2NC) and the UT-WT as reference samples. Transformed seeds were imported and released at the Lytle Ranch Preserve under the US Department of Agricultural Animal and Plant Health Inspection Service (APHIS) permit numbers 07-341-101n, 12-320-103 m, and 18-282-103r.

#### Overview of the datasets

The three datasets, including the measurements from the Arizona field, Utah field, and Jena glasshouse, respectively, are summarized in Fig. 2. Field_AZ included PLs, RILs, and reference (UT-WT). Field_UT included TLs and reference (pRESC2NC and UT-WT). The Glasshouse included PLs, TLs, and reference (both EV lines and UT-WT). These are three independent experiments conducted at different dates & times, as specified in the table. In Field_UT and Glasshouse, the leaves were measured directly on the plants where they grew. In Field_AZ, because of the large number of plants to be measured and because individual plants were not measured repeatedly, the leaves were cut, hydrated and measured in a field station; for details, see section Measurement procedures.

### Optical measurements and calculation of leaf reflectance

#### Equipment

Leaf optical properties were measured using two FieldSpec 4 spectroradiometers (serial numbers 18140 and 18141) coupled with a plant probe and a leaf clip (serial numbers 445 and 455). The FieldSpec® 4 spectroradiometer contains three detectors covering the visible and near infrared (VNIR, 350–1000 nm) to the shortwave infrared (SWIR, 1001–1800 nm and 1801–2500 nm) range of electromagnetic radiation. The VNIR detector is a silicon photodiode array while the two SWIR detectors are thermo-electrically cooled indium gallium arsenide. The spectral resolution is 3 nm at 700 nm and 10 nm at 1400 and 2100 nm. Date are interpolated by the calibrated FieldSpec system to output a value for every nanometer of the spectrum between 350 and 2500 nm. Detectors are covered by order separation filters. The contact probe is a holder with a round glass window allowing radiation from a halogen radiation source to pass through, and contacted by the tip of the fiber-optic cable transmitting radiation to the three detectors, such that the radiation source and detection are on the same side of the sample and both are fixed in position relative to each other and the sample. Randomly distributed, individual fibers of the fiber-optic cable each direct radiation to one of the three detectors and radiation is dispersed by a holographic diffraction grating (ASD Inc, 2010). The leaf clip is a cylinder which can be turned to show either a flat white background or a flat back background and the flat sides can be affixed against the window with a gentle clipping mechanism (ASD Leaf Clip Manual from URL: https://www.azom.com/equipment-details.aspx?EquipID=5411).

#### Measurement procedures

In all cases, leaves were measured at the widest part of the leaf, avoiding the midvein, with the adaxial side of the leaf facing the light source and fiber-optic cable; see further details in the Equipment section. For each sample, the measurements consisted of 20 scans under four conditions in order: 5 scans of the white background reference (WR), 5 scans of the white background reference with the leaf (adaxial side facing the sensor, WRL), 5 scans of the black background reference (BR), 5 scans of the black background reference with the leaf (adaxial side facing the sensor, BRL).

In the Glasshouse, the measurements were performed on leaves at a standardized position directly on plants (+2, rosette, denoting the second fully expanded leaf [46]), and the instrument was adjacent to the growth table (each pot was moved to a cart for measurement). Measurements were conducted on December 13th, 2018, 34 d after sowing, after plants had bolted and had mature stem leaves, but before they had reached their full height or begun to flower. Additional test measurements were also conducted prior to the UV-B treatment; because these did not include the full set of replicates, and as we are not interested here in the specific influence of UV-B light on plants in the glasshouse, those data were neither included nor used for the analysis presented here.

In Field_AZ, plants were bushy. Because of the large number of plants to be measured, we cut leaves off the plants and placed them into individual cups containing a layer of water at the bottom, so that petioles were in water and lamina were not, and measured the cut-off leaves inside the nearby field station. The total time from detachment to measurement did not exceed 2 h. Leaves remained turgid during this period. We note that some water absorption features did significantly differentiate leaf spectra of the reference genotype in this experiment from those in the two other experiments, but these same water absorption features also differed between the Field_UT and Glasshouse experiments, in which all measurements were made with leaves still on the plant (see Results). Due to the different sizes of leaves, we harvested and measured either one or two stem leaves to fill the leaf clip. When measuring two leaves, leaves were placed adjacent to each other to fill the measurement window without overlap or gaps. We selected intact, mature, non-senescent, sun-exposed stem leaves at a similar position (S2–S5, [46] when possible or else a similar position on a side branch). As long as leaves were sufficiently intact to measure (no large holes), we did not exclude damaged leaves if they were generally representative of the condition of leaves on the plant. Each batch included seven 4-plant clusters (Additional file 1: Fig. S1a). Within one batch, RIL and PL leaves, and three of the UT-WT plants were collected and measured. Not all UT-WT plants were measured in the interest of time. Measurements were conducted on 7 contiguous days, from the 15th to the 21st of July 2019 (ca. 87-93 d after sowing), when plants were large and reaching reproductive maturity but the great majority of leaves were still photosynthetically active (non-senescent). This timing was coordinated to follow earlier observations of the same plants [44]. Measurements started shortly after dawn and ending at dusk (ca. 7 am–8 pm). On day one, three batches of 8–17 samples each (including 1–2 Ref samples per batch) were measured; from day two onward, measurements were done on batches of 24 samples (21 RILs/PLs + 3 Ref).

In Field_UT, measurements were conducted on intact, mature, non-senescent leaves at a standardized developmental stage from plants directly in the field (upper rosette or lower stem leaf: 0, S1 or S2 chosen to match developmental stage and avoid damage, [46]; for the leaf position experiment leaves at all three positions were measured within the same plant). The phenology of plants at the Utah site is ca. one month earlier than those at the Arizona site, and experiments in UT were conducted at the end of May 2019 (69–72 days after sowing), at which time plants were in a similar growth stage as in the Glasshouse experiments, although stalk elongation had proceeded further in comparison to the growth stage used in Glasshouse measurements. To compare TLs across different days and measurement times, the same set of plants (TLs, EV) were measured at three different time points (at am on May 24th, at noon on May 27th, at pm on May 25th, 2019). To compare the effects of leaf position, we conducted a separate experiment, where three leaves (old—rosette, intermediate—lower stem, young—mid-stem) were measured within the same plant for a set of UT-WT plants at three different time points (at am on May 25th, at noon on May 26th, at pm on May 24th, 2019, see section Growth conditions and environments). Note that the leaf position experiment was conducted at different times from the TL screening experiment described above, i.e., these two experiments were measured at different time points even within the same day. Twenty measurement scans of the development experiment were missing on the first measurement day (May 24), and thus we excluded all measurements of the development experiment on this day.

#### Raw data processing

We performed raw data processing with R (version 4.3.0, mainly using the packages spectrolab (version 0.0.10) [47] and DescTools(version 0.99.49) [48]. We removed outliers with a three-step approach. In the first step, we visually inspected the plots of different measurement types (white reference, white reference with leaf, black reference, black reference with leaf) and removed samples that clearly did not belong to the measurement type, e.g., measurements that were supposed to be of a white reference but which showed a shape typical of a leaf spectrum were considered to be errors and were removed. In the second step, we applied the Local Outlier Factor (LOF) method [49] to each measurement type. This method is a standard and widely accepted technique for outlier detection and is particularly suited to our dataset. LOF takes into account both the density and distance of data points, providing robust detection amidst variable spectral data across genotypes. Its local approach is particularly important—LOF compares each data point primarily with its neighbors – as certain genotypes, such as TLs, might exhibit spectra that substantially differ from others due to specific gene expression interference, and these differences are not necessarily indicative of outliers. Lastly, after the calculation of reflectance (see section Calculation of leaf reflectance), we performed a final visual inspection. Given the significant impact of outliers on major components of our analysis (coefficient of variation, linear modeling), we combine LOF and visual checks to strictly exclude outliers. The first scan under each condition was systematically removed to avoid potential contamination from previous readings and to ensure stabilization of the signal. When another scan or more than one of the remaining four scans were detected as outliers, the corresponding scan (if only one under each condition) or the corresponding plant (more than one scan under each condition) was removed. Additional file 1: Fig. S2a–c illustrates examples of outliers detected at each step. More details and records can be found in the code “RawDataProcess.R” (see section Availability of data and code). After the raw data processing, in total for Field_AZ, five samples (137, 165, 209, 813, 815) and for the Glasshouse, three scans (73, 403, 554) were removed. For Field_UT, since the same plants were measured three times, to be able to use as much data as possible, we only excluded the sample in the corresponding measurment time in which it was detected as an outlier, and not necessarily in the other two measurment times. For the main experiment, plants 31 and 35 were removed from am measurements, plants 42 and 47 and scans 784, 1361, and 1381 were removed from noon measurements, and plants 21, 42, 47, 59, 61, 69 , and scans 984 and 976 were removed from pm measurements. For the leaf position side experiment, plant 13 leaf 1, and scans 602, 629, 579, 659, 779, and 819 were removed from am measurements, and plant 11 leaf 2, plant 13 leaf 1, and scan 696 were removed from noon measurements. As described in section Measurement procedures, we did not include pm measurements on May 24 of the leaf position experiment because 20 scans were missing.

#### Calculation of leaf reflectance

We analyzed the spectral range from 400–2500 nm because the regions at the beginning (350–399 nm) have a poor signal:noise ratio [9] and are commonly removed from downstream analysis. As detailed in the section Raw data Processing, the first scan was dropped because of the potential influence from previous readings or the opening of the leaf clip. Then the averaged reflectance under each condition was obtained with the mean reflectance of the last four scans (or three scans for a few specific cases in which one of these four scans was an outlier, see section Raw data Processing):

1 $$\begin{aligned} R_c = (R_{c,2} + R_{c,3} + R_{c,4} + R_{c,5}) / 4 \end{aligned}$$

where

2 $$\begin{aligned} c = \left\{ WR, WRL, BR, BRL \right\} \end{aligned}$$

The calculated reflectance of a sample was obtained from the mean of scans above ($R_c$) using the following formula from [50]:

3 $$\begin{aligned} CR = (R_{WR} \cdot R_{BRL} - R_{BR} \cdot R_{WRL}) / (R_{WR} - R_{BR}) \end{aligned}$$

The absolute measurement uncertainty (AU) of a sample was calculated using the formula from [51], which was derived from [50]:

4 $$\begin{aligned} AU_{CR}^2= & {} \left( \frac{BR\left( WRL-BRL\right) }{\left( WR-BR\right) ^2}\right) ^2 \left( \frac{S T D_{WR}}{\sqrt{N}}\right) ^2+\left( \frac{BR}{WR-BR}\right) ^2 \left( \frac{STD_{WRL}}{\sqrt{N}}\right) ^2\nonumber \\{} & {} +\left( \frac{WR\left( WRL-BRL\right) }{\left( WR-BR\right) ^2}\right) ^2 \left( \frac{STD_{BR}}{\sqrt{N}}\right) ^2+\left( \frac{WR}{WR-BR}\right) ^2 \left( \frac{STD_{BRL}}{\sqrt{N}}\right) ^2 \end{aligned}$$

Then the relative measurement uncertainty (RU) of a sample was calculated as the absolute uncertainty divided by reflectance (see Petibon and colleagues, [51]).

5 $$\begin{aligned} RU_{CR}= AU_{CR} / CR \end{aligned}$$

### Analyses of calculated reflectance

We expect that genetic effects on leaf reflectance vary both by experiment and by genotype. Even in the TLs, where genetic differences are targeted and specific, we expect differences among genotypes to affect several regions of the leaf reflectance spectrum due to the multiple physiological effects from silencing target gene expression. Here, we ask to what extent variance in leaf reflectance spectra is affected by growth environment and genetic variation of the measured plants, as well as other experimental factors including measurement time, different numbers of measured leaves (1 or 2), and different leaf positions within the same plant.

#### Principal components analysis

In order to describe variance structure across and within datasets, we conducted principal components analyses (PCA) using the R package factoextra (version 1.0.7). First, we conducted a PCA on the whole dataset to assess the main sources of variance in general, both for all lines together, and for one genotype (UT-WT) only, in order to better understand the magnitude of variance contributed by genotype groups and experimental conditions. We described the variance explained by each principle component, as well as the contribution of variables (leaf reflectance calculated at each wavelength) to each component. Then, we conducted the same analyses on the three datasets separately, in order to characterize the relative contribution of genotype groups to total variance by experiment. More details can be found in the code “Plots_PCA.R” (see section Availability of data and code).

#### Coefficient of variation, and bootstrapping

We furthermore calculated the coefficient of variation (CV) (referred to as spectral variation by Petibon and colleagues, [51]) as an indicator of the variation within or, more often, among groups. When there were unequal numbers of samples in groups, we used bootstrapping to downscale the sample size of the group with the larger number of samples, to the sample size of the smaller group. For each run, we conducted bootstrapping 100 times using the *sample()* function in R. More details can be found in the code “Plots_PCA.R” and “Plots_Models.R” (see section Availability of data and code).

#### Linear regression and linear mixed models

To study the influence of genetic and non-genetic variance on differences in measured leaf reflectance within each environment, we built models within each dataset to determine the effects of different factors, such as measurement of test lines (PLs, RILs, and TLs) versus reference lines (UT-WT and EV), or measurement at noon or in the afternoon (pm) compared with measurement in the morning (am) (Table 2). Based on the experimental setting and research questions for each dataset, we built different models including one-way ANOVA, linear regression (with *post-hoc* tests when further pairwise comparisons were needed using a Tukey adjustment), and mixed models where random effects due to repeated measurements over different days on the same plant were taken into account. We used the following R packages to conduct the regression, select the best-fitted models, and generate figures: lme4 (version 1.1-31, [52]), nlme (version 3.1-160, [53]), MASS (version 7.3-58.1, [54]), emmeans (version 1.8.2, [55]), and tidyverse (version 1.3.2, [57]). We used R package report (version 0.5.5, [56]) to generate formatted reports of models. In the reports, effect sizes were labeled following Field’s recommendations [58].

For model selection, we first determined the main effects based on our knowledge and experimental settings. For each dataset, we selected 4 wavelengths that contributed most to the first 4 PCs of the corresponding dataset. We then used Akaike’s Information Criterion (AIC, [59]) to compare different models, and selected the model (with main effects) that fit best in the majority of these 4 wavelengths. We then used forward stepwise selection, starting from the selected model with main effects, and with the full model including all interactions among these main effects, to determine the final model. When the number of main effects was equal to or smaller than two, we included the interactions and compared the models directly for simplicity. More details can be found in the script Plots_Models.R (see section Availability of data and code).

**Table 2:** Models built in this study

Model	Experiment	Variables	Random effect	n	Figure
1	Field_AZ, Field_UT, Glasshouse	Experiment (UT-WT only)		119	4
2	Field_AZ	Genotype group, measurement time, batch, leaf number		779	6a, b, 8c, Additional file 1: S4a, c, 9a,
3	Field_AZ	measurement time, batch, leaf number		99	8d, 9b, Additional file 1: S4b, d
4	Glasshouse	Reference line		13	Additional file 1: S3a
5	Glasshouse	Genotype group		39	7d
6	Glasshouse	Genotype		28	7c
7	Field_UT	Measurement time, genotype	1 $\mid$ Plant ID	149	7a, 8a, b
8	Field_UT	Measurement time, leaf position	1 $\mid$ Plant ID	39	9a, b

The final models used in this study are summarized in Table 2. We conducted one-way ANOVA on the UT-WT samples across the three experiments (Table 2 Model 1) using the formula Reflectance $\sim$ Experiment. For Field_AZ, we fitted a linear model (estimated using ordinary least squares regression, OLS) to predict reflectance with genotype groups (Table 2, Model 2), measurement time, batch, and leaf numbers: formula, Reflectance $\sim$ GeneGroup + Mtime + Batch + Leaf_Num, and a linear model contained only UT-WT samples to predict reflectance (Table 2, Model 3) with measurement time, batch, and leaf numbers: formula, Reflectance $\sim$ Mtime + Batch + Leaf_Num. In Glasshouse, we conducted one-way ANOVA on the reference samples (UT-WT, EV1, EV2) using the formula Reflectance $\sim$ Reference line (Table 2, Model 4). We then divided the dataset into two subsets (PLs + UT-WT, TLs + EV) and selected two models on each subset. Table 2, Model 5 contained PLs and UT-WT samples, and the genotype group as the only predictor, thus it was the same as a one-way ANOVA with the formula Reflectance $\sim$ GenoGroup. Table 2, Model 6 contained TLs and EV samples. Here we also fit a linear model to predict Reflectance with GeneGroup (formula: Reflectance $\sim$ GeneGroup). In Field_UT, we built two models for the line-screening experiment and leaf position experiment separately, both accounting for the repeat-measure design. Table 2 Model 7 contained TLs and EV (screening experiment). We fit a linear mixed model (estimated using REML and nlminb optimizer) to predict reflectance with measurement time, genotypes, and their interaction (formula: Reflectance $\sim$ 1 + Mtime * Genotype). The model included plant ID as a random effect (formula: $\sim$1 $\mid$ Plant_ID). Table 2 Model 8 corresponds to the leaf position experiment. We fitted a linear mixed model to predict Reflectance with GeneGroup, measurement time, and leaf ID (formula: Reflectance $\sim$ 1 + GeneGroup + Mtime + leaf). The model included Plant_ID as random effect (formula: $\sim$1 $\mid$ Plant_ID).

#### P-value adjustment for multiple testing

We adjusted p-values for the multiple tests conducted across wavelengths with the function p.adjust() in R using method “BY” based on the study of Benjamini and Yekutieli [60]. This controls the false discovery rate: the expected proportion of false discoveries amongst the rejected hypotheses, which is commonly used e.g. for the analysis of transcriptomic or metabolomic datasets having similar or more dimensions compared to our spectroscopy dataset of ca. 2 000 wavelength “features” [61].

## Results

### Model performance

Here, we report model test results for wavelength 500 nm to indicate total variance explained, and variance structure, for the various linear models used across the dataset (Table 2). In linear models and linear mixed models, the standardized parameters were obtained by fitting the model on a standardized (normalized) version of the dataset. The 95% Confidence Intervals (CIs) and p-values were computed using a Wald t-distribution approximation. Adjusted p-values across all wavelengths are presented alongside supporting analyses (PCA, CV comparison) in the following sections.

We conducted one-way ANOVA on the UT-WT samples across the three experiments (Table 2 Model 1), which suggested that the main effect of experiment was statistically significant and large (F_2, 142_ = 18.63, p < 0.001; Eta^2^ = 0.21, 95% CI [0.11, 1.00]). We also used one-way ANOVA for the comparison of UT-WT, EV1, and EV2 samples in the Glasshouse (Table 2 Model 4). This ANOVA (formula: Reflectance $\sim$ Genotype_ID) indicated that the main effect of Genotype_ID was statistically non-significant and small when comparing these three reference genotypes (F_2, 10_ = 0.27, p = 0.769; Eta^2^ = 0.05, 95% CI [0.00, 1.00]).

For Field_AZ, we fit two linear models. (One (Table 2 Model 2), built to predict reflectance with genotype groups, measurement time, batch, and leaf numbers, explained a statistically significant and moderate proportion of variance (R^2^ = 0.17, F_39, 739_ = 3.76, p < 0.001, adj. R^2^ = 0.12). The model’s intercept, corresponding to UT-WT plants in the morning measurement, batch 1, one leaf, was at 0.09 (GeneGroup = UT-WT, Mtime = am, Batch = 1, Leaf_Num = 1, 95% CI [0.08, 0.11], t_739_ = 11.77, p < 0.001). The second model (Table 2 Model 3) was designed to explore the same non-genetic effects with only the UT-WT samples. This model explained a statistically not significant but substantial proportion of variance (R^2^ = 0.46, F_36, 62_ = 1.45, p $=$ 0.098, adj. R^2^ = 0.14). The model’s intercept, corresponding to morning measurement, batch 10, one leaf, was at 0.09 (Mtime = am, Batch = 1, Leaf_Num = 1, 95% CI [0.08, 0.10], t_62_ = 17.37, p < 0.001).

In the Glasshouse, we divided the dataset and selected two models on each subset. Table 2 Model 5 contained PLs and UT-WT samples, and genotype group was the only predictor, thus it was the same as one-way ANOVA. The ANOVA suggested that the main effect of genotype group was statistically significant and large (F(_1, 37_ = 10.93, p = 0.002; Eta^2^ = 0.23, 95% CI [0.06, 1.00]). Table 2 Model 6 contained TLs and EV samples. We fit a linear model to predict reflectance with genotype group, and this explained a statistically not significant but substantial proportion of variance (R^2^ = 0.35, F_7, 20_ = 1.54, p = 0.211, adj. R^2^ = 0.12). The model’s intercept, corresponding to GeneGroup = EV, was at 0.07 [95% CI (0.07, 0.08), t_22_ = 49.92, p < 0.001].

In Field_UT, we built two models for the line screening experiment and the leaf position experiment separately. Table 2 Model 7 contained TLs and EV samples. We fit a linear mixed model to predict reflectance with measurement time, genotypes, and their interaction, including plant ID as a random effect due to the repeated measures design. The model’s total explanatory power was substantial (conditional R^2^ = 0.19) and the part related to the fixed effects alone (marginal R^2^) was 0.12. The model’s intercept, corresponding to Mtime = am, Genotype = EV, was at 0.08 (95% CI [0.07, 0.09], t_94_ = 18.74, p < 0.001). Table 2 Model 8 corresponded to the leaf position experiment. We fit a linear mixed model to predict reflectance with measurement time, and leaf ID, including plant ID as a random effect. The model’s total explanatory power was substantial (conditional R^2^ = 0.25) with the the part related to the fixed effects alone (marginal R^2^) equal to 0.13. The model’s intercept, corresponding to GeneGroup = UT_WT, Ctime = am, leaf = 2, was at 0.11 (95% CI [0.09, 0.12), t_29_ = 18.83, p < 0.001].

### Total variance across the three experiments

**Fig. 3:**
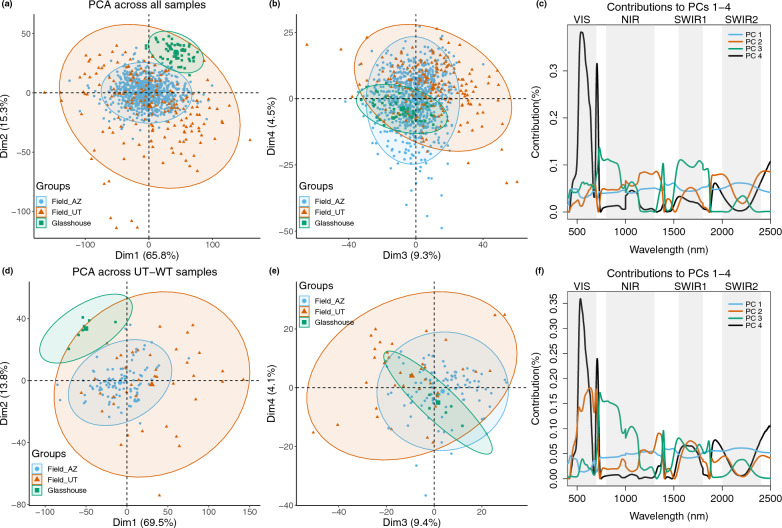
Principle components analyses (PCA) across all three datasets indicate that experimental setting determines variance structure. **a**, **b** PC1-4 of PCA across all samples in the three datasets. Each blue circle is a sample measured in Field_AZ, each orange triangle is a sample measured in Field_Utah, and each green square is a sample measured in the Glasshouse. This color scheme is retained in further figures, unless stated. Shaded regions indicate 95% confidence intervals. **c** Contribution of reflectance calculated at each wavelength to the first 4 PCs. Blue: PC1, orange: PC2, green: PC3, grey: PC4. **d**, **e** PC1–4 of PCA across the three datasets using only UT-WT samples; **f** contribution of wavelengths to the first 4 PCs. The color and shading scheme of (**d**)–(**f**) is the same as in (**a**)–(**c**)

We conducted principal component analysis (PCA) across all three datasets (Fig. 3a–c). The Field_UT dataset (orange) had the largest range of variance and the Glasshouse dataset (green), the smallest. The two field-collected datasets revealed overlapping distributions along PC1 and PC2, with the Field_UT dataset being more widely scattered and the Field_AZ, more tightly clustered. The Glasshouse dataset was clearly separated from the Field_AZ dataset along PC2, but overlapped along PC1. The two field-collected datasets have a more similar range of variance along PC3 and PC4, where Field_AZ dataset was distributed within the range of Field_UT along PC3, and partly separated along PC4. The Glasshouse dataset was distributed within the range of the two field-collected datasets along PC3 and PC4. Together, the first four components explained ca. 95% of total variance. Thus, we examined the contributions of wavelengths to these components (Fig. 3c).

Measured wavelengths contributed almost evenly to PC1. PC2 was dominated by wavelengths in the near-infrared (NIR) especially from ca. 1100–1400 nm, indicative of cell structure, and water absorption bands between SWIR1 and SWIR2 and the wavelengths following SWIR2, with relatively large contributions from the VIS range ca. 550–700 nm and in the SWIR1. PC3 was dominated by wavelengths at the red edge and NIR from 750–1000 nm, the SWIR1, and a peak at ca. 1400 nm in the water absorption band between NIR and SWIR1. VIS wavelengths contributed most to PC4. The overall distribution of experiments along the first 4 PCs was similar when analyzing only reference (UT-WT) samples (Fig. 3d, e), except that PC2 was instead dominated by VIS, the water band between NIR and SWIR1 and the SWIR1, indicating that experimental differences, rather than different compositions of genotypes in each experiments, explained a large proportion of the variance structure across the three datasets.

**Fig. 4:**
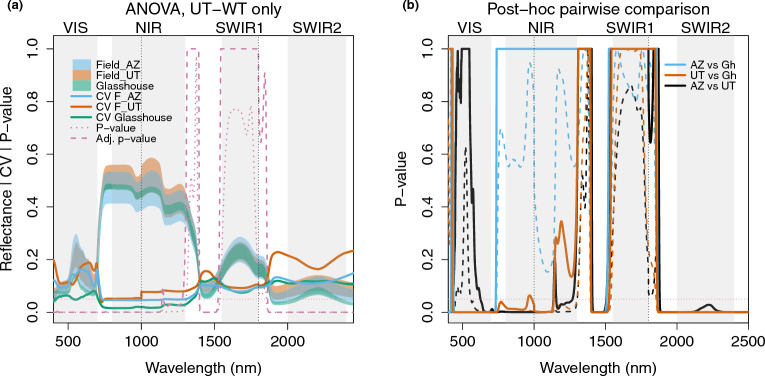
Comparing leaf spectra of Reference (UT-WT) samples reveals widespread effects of experimental setting on the VIS, NIR and SWIR2, and water absorption bands, and a common effect of field versus glasshouse settings on visible wavelengths. **a** Each shaded region in blue, orange, or green indicates the range of calculated reflectance for all UT-WT samples in the corresponding experiment (Field_AZ, Field_UT, Glasshouse). Each solid colored line is the coefficient of variation of all UT-WT samples in the corresponding experiment. The pink dotted line shows the p-values from a one-way ANOVA across all UT-WT samples with experiment as the factor. The pink dashed line shows the p-values after Benjamini-Yekutieli adjustment. Horizontal pink dotted lines indicate a p-value of 0.05, which is commonly used as a threshold for statistical significance. **b** P-values from Tukey *post-hoc* tests for pairwise comparisons following the ANOVA: blue, Field_AZ vs. Glasshouse; orange, Field_UT vs. Glasshouse; black, Field_AZ vs. Field_UT. Dashed lines: original p-values from *post-hoc* tests; solid lines: Benjamini-Yekutieli-adjusted p-values

We furthermore conducted Analysis of Variance (ANOVA) on the reference (UT-WT) samples in the three experiments for leaf reflectance at each wavelength across 400 nm-2500 nm (Table 2 Model 1), followed by Tukey *post-hoc* pairwise tests and using the Benjamini-Yekutieli adjustment for multiple testing, to indicate non-genetic variation (Fig. 4). We used the coefficient of variation (CV) of the calculated reflectance (section Coefficient of variation, and bootstrapping) to represent the variation among different groups of plants. Such variation may result from several factors, including growth environment, measurement time and methods, as summarized in Fig. 2. We investigate these factors in more detail in the following sections.

The differences across all reference (UT-WT) samples were greatest across the VIS, NIR and SWIR2 parts of the spectrum, including some bands between the NIR and SWIR1, and between the SWIR1 and SWIR2 regions, dominated by water absorption, as shown in Fig. 4a. Water absorption differed among environments regardless of whether measurements were made on-plant (Glasshouse, Field_UT) or on hydrated cut leaves (Field_AZ), as seen in Fig. 4b. When comparing glasshouse measurements to either field measurement, spectra varied most in the VIS and into the red edge, water absorption bands and the SWIR2. Interestingly, Field_UT samples also differed strongly from Glasshouse samples in the NIR, while Field_AZ samples did not, although leaf morphology in the AZ experiment was distinct from the Glasshouse and Field_UT experiments (Additional file 1: Fig. S1d). Samples from the two field experiments differed from each other most in the red edge between the VIS and the NIR, water absorption bands, and in the SWIR2 region (Fig. 4b). Thus, the common difference between the glasshouse and field experiments was in the VIS and in the water absorption bands in the SWIR.

### Total variance within each experiment

**Fig. 5:**
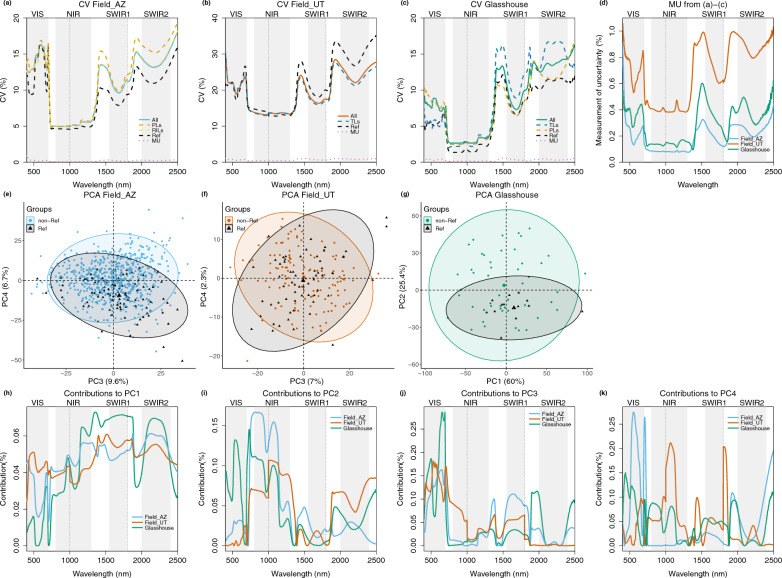
Genotype groupings influenced variance structure differently within each of the three experiments, with visible and SWIR absorption bands contributing most consistently to the PCs that best distinguish genotype groups. **a**–**c** Coefficient of variation for spectra of genotype groups compared to relative measurement uncertainty within each experiment. The measurement uncertainty of the three experiments is separately shown in (**d**) for comparison. **e**–**g** Distribution of samples along PCA dimensions best distinguishing different genotype groups: PC3 and PC4 for the field experiments (**e**, **f**), and PC2 (shown versus PC1) for the glasshouse experiment (g). Shaded regions show 95% confidence intervals for each group, i.e. reference samples (UT-WT, EV) versus non-reference samples (PLs, RILs, TLs). **h**–**k** Contribution of reflectance at each wavelength to the first 4 PCs in each dataset

We then investigated variance within each of the three datasets individually. Figure 5a–c shows the coefficient of variation for different groups and relative measurement uncertainty in each dataset. For the Field_AZ and Glasshouse datasets, the reference samples were the least variable group across most bands, but not for the Field_UT dataset. Measurement uncertainty of three datasets is shown separately in Fig. 5d, and was mostly smaller than 1% across the analyzed wavelengths. This corresponds to less than 10% of spectral variation within each experiment, which ranged from ca. 5–20% in the Field_AZ and Glasshouse experiments and from 10–40% in the Field_UT experiment. Figure 5e–g shows the distribution of samples along PC3 and PC4 in the two field datasets, or PC1 and PC2 in the Glasshouse dataset, which are the principle components that best distinguished the reference and non-reference groups in each experiment (see distributions along other PCs in Additional file 1: Fig. S3). In all three datasets, the first PC explained more than half of the variance, and the first four PCs explained over 95% of the total variance (Additional file 1: Fig. S3). Clusters comprising either reference samples (UT-WT and/or EV) or non-reference samples (PLs, RILs, and/or TLs, see Fig. 2) mostly overlapped along PCs 1 and 2 in field datasets, and differed more along PCs 3 and 4 in their region of overlap, especially for Field_AZ (Fig. 5e). As for the Glasshouse dataset, reference samples clustered more tightly than non-reference samples along PCs 1 and 2 (Fig. 5g), while the two groups mostly overlapped along PCs 3 and 4. Figure 5h–k shows the contribution of reflectance at each wavelength to the first 4 PCs.

PC1 explained 60–71% of variance per dataset (Additional file 1: Fig. S3). The region from 400–500 nm (violet to green light) contributed about five times as much to PC1 for the two field datasets, as for the Glasshouse dataset. Otherwise, there was a similar contribution of different wavelengths to PC1, except for the range around 500–700 nm (green to red light) and the red edge, which contributed least to PC1 in Glasshouse and Field_AZ (Fig. 3d). From ca. 800 nm onward (NIR), contributions of individual wavelengths to PC1 ranged from about 0.03%, to about 0.08% (which was the maximum contribution of any wavelength to PC1), with longer wavelengths tending to contribute more in all of the individual datasets (but with dips in the water absorption bands).

PC2 explained 11–25% of total variance in each dataset (Additional file 1: Fig. S3) and was generally dominated by contributions in the NIR, but with more variable patterns. In the Glasshouse – where PC2 explained 25% of total variance, and where reference samples clustered more tightly than non-reference samples along PC2 – the region from 400 nm to ca. 600 nm (violet to orange light) contributed about six times as much as it did in the field datasets. This corresponds to higher spectral variation in that region for the PLs than for the reference genotypes in the Glasshouse dataset (Fig. 5c). For Field_AZ, where PC2 explained only 11% of total variance, the NIR region of greatest reflectance from ca. 800–1100 nm contributed about twice as much to PC2, as it did for the other two datasets. For Field_UT, where PC2 explained 16% of total variance, the SWIR2 region and neighboring water absorption bands contributed about twice as much to PC2 as for Field_AZ, with the water absorption bands contributing relatively more. The pattern of contributions to PC2 in the SWIR was similar for the Glasshouse and the Field_UT datasets, and the magnitude of contribution was only slightly lower in the Glasshouse.

The entire VIS region dominated contributions to PC3 across all three datasets (explaining 7–10% of total variance per dataset, Additional file 1: Fig. S3), but this contribution was most pronounced for Glasshouse from ca. 650–750 nm (red wavelengths), followed by the two field datasets.

Finally, PC4 explained 2–7% of total variance per dataset (Additional file 1: Fig. S3). For Field_AZ, where PC4 explained 7% of total variance, VIS wavelengths, especially from ca. 500–600 nm (green-orange) and 700–750 nm (red light), contributed most, together with the water absorption bands between SWIR1 and SWIR2, and wavelengths near 2500 nm. In Field_UT, where PC4 explained only 2% of total variance, the greatest contributions came from wavelengths in the NIR region (from ca. 1000–1100 nm), and part of the water absorption band following SWIR1, with substantial contributions in the red and other NIR and SWIR1 wavelengths and part of the water absorption band between NIR and SWIR1. For the Glasshouse, where PC4 explained only 3% of total variance, the greatest contributions came from several regions in the VIS (ca. 400–600 nm, blue to orange), NIR (red edge to ca. 1100 nm with a dip at ca. 1000 nm), SWIR1, and from the same absorption bands in the SWIR which also loaded onto PC4 for Field_AZ.

### Genotypic effects on spectral variation

**Fig. 6:**
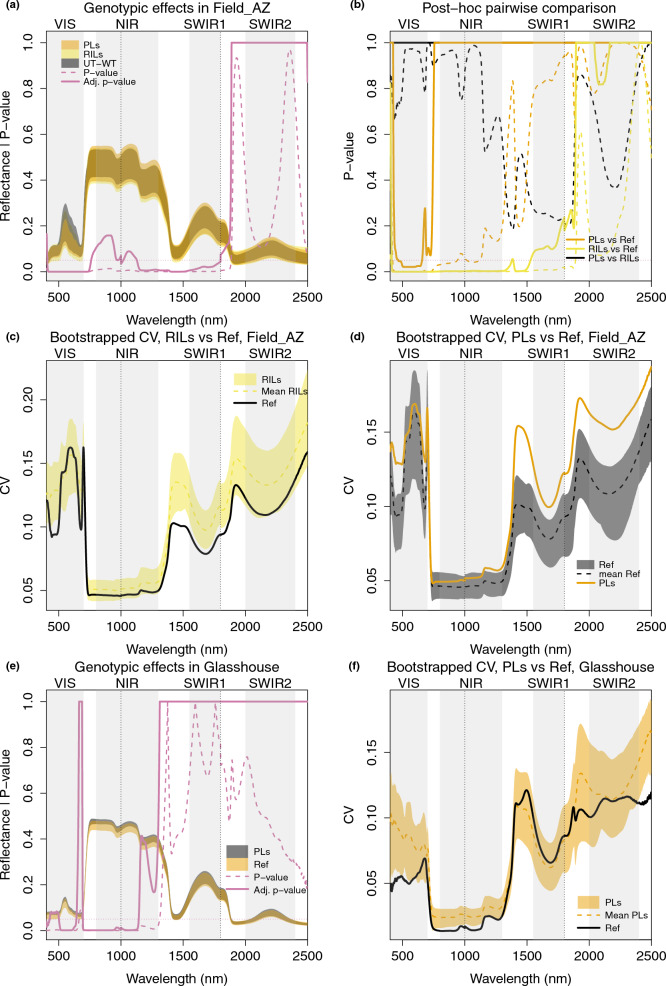
PLs and RILs are best distinguished from reference genotypes (UT-WT, EVs) by visible and near-infrared reflectance peaks, in the field and in the glasshouse. **a** ANOVA across reflectance of PLs, RILs, and reference (UT-WT) samples in Field_AZ. Each colored shade in orange, yellow, or grey is the range of reflectance of all samples from the corresponding genotype group (PL, RIL, UT-WT). The pink dashed line is the p-value of ANOVA on all samples in three groups, and the pink solid line is the p-value after adjustment. **b** Adjusted p-values from *post-hoc* tests for pairwise comparison. Orange shows the comparison between PLs and UT-WT, yellow between RILs and UT-WT, and black between PLs and RILs. **c** The coefficient of variation (CV) of RILs and UT-WT samples. The yellow shaded area indicates the range of bootstrapped (100 times) CV results for RILs and the dotted yellow line indicates the mean of the bootstrapped results. The black line shows the CV of the UT-WT samples. **d** The CV of PL and UT-WT samples. The grey shaded area indicates the range of bootstrapped (100 times) CV results for the UT-WT samples and the dotted black line indicates the mean of the bootstrapped results. The orange line shows the CV of the PL samples. **e** ANOVA across reflectance of PLs and reference(UT-WT, EVs) samples calculated from Glasshouse measurements. Interpretations of colors and lines are the same as in (**a**). **f** The CV of PLs and reference samples. The orangle shaded area indicates the range of bootstrapped CV results for PLs (100 times) and the dashed line indicates the mean of the bootstrapped results. The black line shows the CV of the reference samples

To study the genotypic effects on variance within each experiment, we first investigated features distinguishing populations in the MAGIC forward genetics experiment. We conducted comparisons separately for Field_AZ and Glasshouse measurements (Fig. 6).

In the larger experiment conducted in Field_AZ, we compared three groups of genotypes: the MAGIC RILs, MAGIC PLs, and the interspersed UT-WT reference, using linear model 2 in Table 2. The difference among all three groups was significant from 400 nm to 1800 nm except for ca. 800–1100 nm, including the VIS, NIR, and SWIR1 regions (Fig. 6a): differences starting from the end of the red edge at 760 nm, to the middle of the NIR region at 1000 nm, and from 1018 nm to 1127 nm, became non-significant after adjustment. In pairwise comparisons (Fig. 6b), these were the regions that differed significantly between RILs and the reference plants, whereas PLs differed from the reference only in the VIS region. PLs and RILs did not significantly differ in pairwise comparisons (Fig. 6b). Figure 6c, d shows the comparisons of CV. Overall, the spectral variation amongst both the RILs and the PLs was greater than that amongst the reference samples across most spectral bands, even when using bootstrapping to control for different total sample numbers, and especially outside of the NIR region of highest absolute reflectance values (Fig. 6c, d).

The smaller glasshouse experiment included the PLs compared to a set of reference lines comprising both UT-WT and two EV genotypes, which did not differ significantly from each other (Additional file 1: Fig. S4). In an ANOVA (Table 2 model 4), the greatest differences between the glasshouse-measured PLs and reference samples were in the VIS as well as the NIR regions, at specific wavelengths (400–427 nm, 502–641 nm and 694–1142 nm, Fig. 6e). The differences in total within-group variation were less pronounced in the glasshouse, especially when controlling for different sample numbers, but were clearest in the VIS and NIR regions (Fig. 6f).

**Fig. 7:**
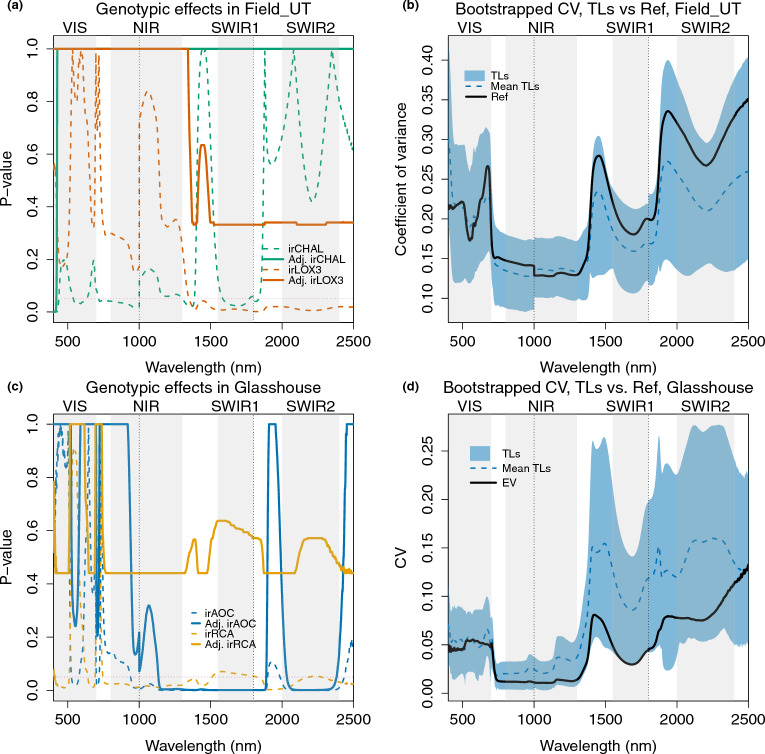
Reflectance spectra of irCHAL differed significantly from the reference genotype (EV) at the beginning of the VIS in the field, and the irAOC line differed significantly from EV in infrared wavelengths in the glasshouse, corresponding to generally greater variation among the TLs in the infrared in the glasshouse, but not in the field. **a** TLs compared with EV in mixed models in Field_UT. Each dashed colored line indicates the p-value of the corresponding TL—green: irCHAL, vermilion: irLOX3 – compared with EV in the mixed model, without adjustment for testing multiple wavelengths. The solid colored line shows the adjusted p-value of the TL versus EV. All other comparisons of EV with transgenics become 1 across all wavelengths after adjustment and can be found in Additional file 1: Fig. S4. (**b**) The coefficient of variation (CV) of TL and EV samples in Field_UT. The blue shaded area indicates the range of bootstrapped (100 times) CV results for TLs and the dashed blue line indicates the mean of the bootstrapped results. The black line shows the CV of the EV samples. (**c**) TLs compared with EV in the linear model in the Glasshouse. Each dashed colored line indicates the p-value of the corresponding TL—blue: irAOC, orange: irRCA—compared with EV in the linear model without adjustment. The solid colored line shows the adjusted p-values. **d** The coefficient of variation (CV) of TLs and EVs samples in the Glasshouse. Interpretations of colors and lines are the same as in (**b**)

We then compared reflectance of samples from the different genotypes in the reverse genetics experiments using transgenic lines. We again conducted comparisons separately for Field_UT and Glasshouse measurements. In Field_UT, we built a mixed model as summarized in Table 2 (model 7). In the Glasshouse, we used linear model 6 in Table 2. In Fig. 7a and c, each dashed colored line indicates the p-values of the corresponding TL compared with EV, prior to correction for multiple testing across wavelengths. Following correction, none of the TLs differed significantly from EV in the field, and in most cases, corrected p-values were equal to 1 across the spectrum, except for irCHAL and irLOX3 in Field_UT, and irAOC and irRCA in the Glasshouse. We therefore only showed the results of these lines in Fig. 7a and c. The full results are shown in Additional file 1: Fig. S4b, c. The CVs (Fig. 7b) were generally larger in Field_UT than in the other experimental settings (Field_AZ, Glasshouse). Surprisingly, the EV group showed a similar or higher CV as the TLs group in Field_UT, indicating larger within-genotype than between-genotype effects on variance in this experiment. In Field_UT, the spectra of one TL, irCHAL, showed significantly different patterns from those of EV after p-value adjustment for multiple testing across wavelength 400–420 nm in the beginning of the VIS range. In the glasshouse, irAOC – like irLOX3, deficient in jasmonate hormones – showed significantly different patterns across wavelengths 1135–1889 nm and 2034–2381 nm in the NIR and SWIR ranges (7c). The CV of TLs tended to be greater than the CV of EV plants grown in the glasshouse (Fig. 7d).

### Other within-experiment effects on spectral variation

#### Effects of measurement time

**Fig. 8:**
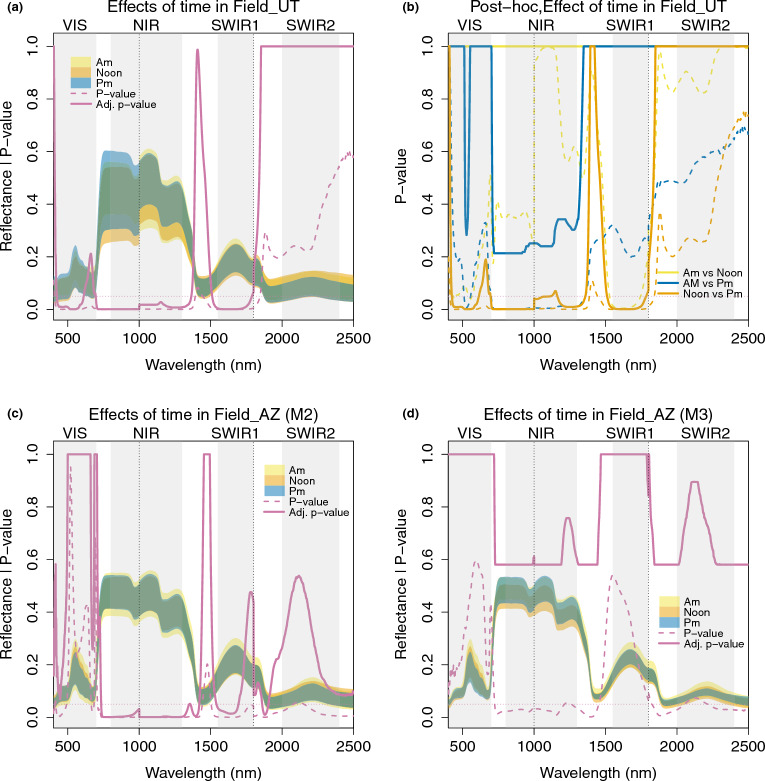
Time of day of measurement effects on leaf reflectance. **a** Effects of measurement time in the mixed model in Field_UT. Shaded areas indicate the range of reflectance for samples measured at am (yellow), noon (orange), and pm (blue). The dashed pink line shows the p-value of the efect of time, and the solid pink line shows the adjusted p-values. **b** P-values from the post-hoc pariwise comparison. The dashed yellow line shows the p-values of am vs. noon measurements. blue line am vs. pm, orange line noon vs. pm. The solid colored lines indicate the adjusted p-values. **c** and **d** Effects of measurement time in the linear model 2 (with all samples) and 3 (with only UT-WT samples) in Field_AZ. The colors and lines have the same interpretation as in (**a**)

In Field_UT, the experiment was designed to broadly assess repeatability over time, and thus the same plants were measured three times, at three different time points (see section Growth conditions and environments). The effects of measurement time were assessed with the mixed model 7 in Table 2. Figure 8a shows the range of reflectance values by time point, as well as the p-values of the effect of time, before and after adjustment for multiple testing. The effect of time was significant in multiple ranges, including in the VIS (424–611 nm), from the red edge to NIR (696–1364 nm), and SWIR1 (1511–1788 nm). Figure 8b shows the post-hoc pairwise comparison on the effect of time. Comparing noon and am measurements, all p-values were 1 after adjustment. The difference between pm and am was a bit larger, and adjusted p-values were less than 1 at some bands in the VIS, around the red edge (671–700 nm), and the whole NIRrange, but never approached the threshold for significance. However, there was a large difference between noon and pm measurements, with this comparison showing very similar p-values with the total effect of time in Fig. 8a, indicating that the difference between noon and pm contributed most to the significant overall effects of time in this dataset.

In Field_AZ, measurements were done in batches to include RILs regularly interspersed with plants of the reference genotype (UT-WT) and the PLs were randomly distributed among the RIL samples, corresponding to the blocked and randomized planting design. These measurements were conducted over 7 consecutive days between dawn and dusk, moving across the field plot from west to east. We divided the daily batches into the same three time groups as for Field_UT (see section Growth conditions and environments). Although the genotype composition of the plants was randomized across measurements (excepting the consistent and regular inclusion of the UT-WT reference genotype), not every one of the ca. 350 genotypes could be measured within each measurement time window, and some genotype group variation thus likely contributed to the other variables in Model 2. To obtain a better estimation of the effects of time, leaf number, and batch, we therefore constructed an additional Model 3 using only the UT-WT samples. Fig. 8c and d show the results of time effects from Model 2 and Model 3 in Table 2. The corresponding post-hoc pairwise comparisons can be found in Additional file 1: Fig. S5a, b. We observed significant time effects in Model 2, and the significance regions were very similar to those identified for Field_UT (see 8a), although the VIS range was not significant, and patterns around the latter part of SWIR1 differed. In contrast, these time effects were not significant in Model 3, although prior to adjustment for multiple testing, the p-values showed a similar pattern and reached the significance threshold at NIR and some water bands, as well as the second half of SWIR2. Similarly, we found a strong batch effect (Additional file 1: Fig. S5c) in Model 2 which was significant across almost all wavelengths (except 1898–2001 nm at the water absorption region), even after p-value adjustment. In contrast, the adjusted p-values for the batch effect all became 1 in Model 3. Together, this indicates that the significant time and batch effects observed in the overall analysis of the Field_AZ dataset are driven by genetic variation among the PLs and RILs, which is not present in the UT-WT samples. This highlights the importance of considering genetic variation when assessing non-genetic effects (time, leaf number, and batch in our case) on leaf reflectance measurements.

#### Leaf number and leaf position

**Fig. 9:**
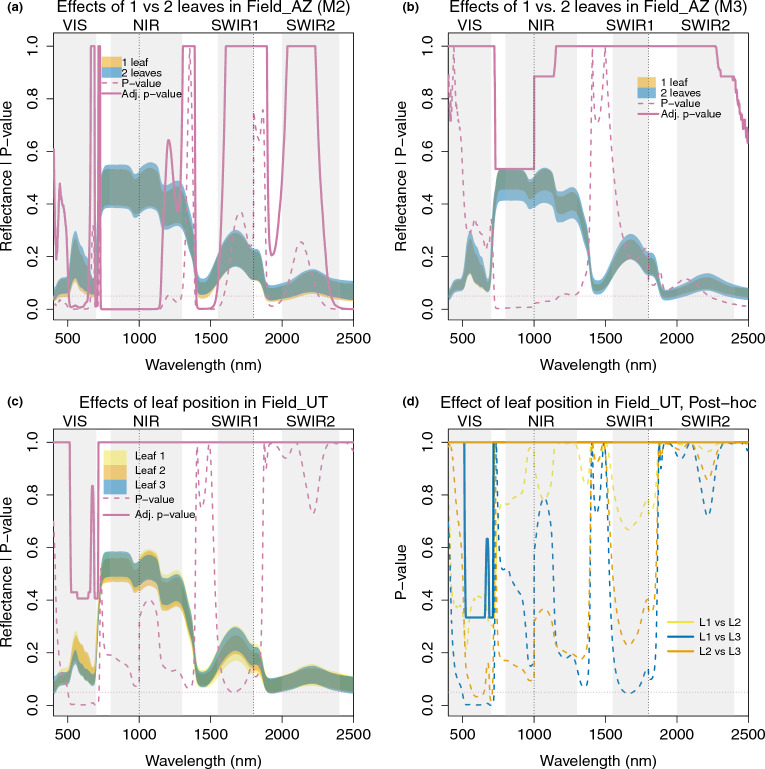
Leaf number required to fill measurement window, and leaf position corresponding to leaf age, affected reflectance at different wavelengths. Effects of number of leaves (one or two) used for reflectance measurements in linear model: **a** Model 2 (with all samples), **b** Model 3 (with only UT-WT samples) in Field_AZ. Shaded areas indicate the range of reflectance for samples measured with 1 leaf (light orange), versus 2 leaves placed side-by-side to form one layer (blue), which was done when needed to fill the measurement window in Field_AZ due to leaves being generally narrower in that experiment. The dashed pink line shows the p-value for 1- vs. 2-leaf measurements. The solid pink lines indicate adjusted p-values. **c** Effects of leaf position in the linear model in Field_UT. Shaded areas indicate the range of reflectance for yellow: younger (Leaf 1), orange: intermediate (Leaf 2), and blue: older (Leaf 3) leaves. The dashed pink line shows the p-value for effects of the leaf position, solid pink line after adjustment. P-values from the post-hoc pariwise comparison. The dashed yellow line shows the p-values of Leaf 1 and Leaf 2 measurements, blue line Leaf 1 vs. Leaf 2, orange line Leaf 2 vs. Leaf 3. Solid colored lines are the p-values after adjustment

In Field_AZ, the plants were bushy and varied morphologically across the field plot. For between-genotype comparisons, this was accounted for by the blocked and randomized design. However, as a result, some plants did not have mature, non-senescent, sunlit leaves at a comparable height which were also large enough to fill the measurement window of the spectroradiometer. In such cases, we conducted the measurement placing two leaves next to each other, side-by-side, in order to fill the measurement window with lamina, without overlapping but also avoiding gaps between leaves. In linear Models 2 and 3 described in Table 2, we included leaf number as a predictor variable. Figure 9a and b shows the resulting p-values. For Model 2, which included all genotypes measured in Field_AZ, the number of leaves (1 or 2) significantly affected reflectance at 508–641 nm, 693–707 nm, 731–1148 nm, 1400–1539 nm and 2344–2500 nm after adjustment: mostly in portions of the VIS and NIR, and in one water absorption region. In Model 3, which included only the reference UT-WT genotype, the effects of leaf number were not significant following p-value adjustment, but were strongest in the NIR and the second half of SWIR2. The selection of two leaves versus one leaf for measurement was dependent on leaf size as described in the section Measurement procedures, and could have affected some genotypes more than others.

In Field_UT, we measured three leaves (older, intermediate, younger) within the same plant for a set of UT-WT plants in order to determine the spectral variation between older and younger leaves at standardized positions (see section Growth conditions and environments). This experiment include 39 UT-WT samples (3 leaves/plant from 13 UT-WT plants). We tested the influence of leaf position and measurement time, controlling for plant ID in a mixed model (Table 2, model 8). Figure 9b and c show the reflectance, resulting p-values from this model, and the post-hoc compairwise comparison. For both comparisons between rosette (older) vs lower stem (intermediate), and younger stem (younger) vs lower stem (intermediate), p-values became 1 across all wavelengths after adjustment. There were larger differences between younger stem (younger) and rosette (older) leaves in the latter part of VIS, which largely contributed to the total leaf position effect shown in Fig. 9a. Leaf position “intermediate” is equivalent to the leaf position used for the screening experiment in Field_UT and representative of the developmental stage for leaves chosen in all three experiments.

## Discussion

We investigated the use of optical spectroscopy to assess plant genetic variation using 360 inbred lines of the wild tobacco *Nicotiana attenuata*, in a set of three experiments under different (glasshouse and field) conditions, and using different types of genetic variation, from natural variants, to a genetic mapping population derived from those variants, to transgenic lines. Plant composition, time course of measurement, aspects of the measurement method (on-plant or off, specific leaf developmental stages) and experimental setting varied within and across the three experiments (see Growth conditions and environments for details, and the summary table in Fig. 2b). These factors, some introduced by design and others by necessity, limit the comparisons we can make, but allow us to study effects of common experimental factors on our ability to detect differences among a large and controlled set of natural and transgenic genotypes, and to compare measurements across experiments.

We first analyzed the overall variance structure among and within experiments using principal component analysis (PCA), and found that experimental setting (Field_AZ, Field_UT, Glasshouse) had large effects on total variance. We found an influence of genetic variation on clustering along PC2 (explaining up to 25% of variance), PC3 (explaining ca. 10% of variance), or even PC4 (explaining up to 7% of variance), depending on the experiment (Fig. 5d–f). We then analyzed genetic and non-genetic sources of variance within each experiment separately. We compared the spectrally resolved coefficients of variation for genotype groups while controlling for different sample sizes, and we built linear regression and mixed models to study the effects of different factors across leaf spectra. We described the magnitude of variance resulting from these factors and the wavelength regions affected. The results can help to design future studies using spectroscopy to assess plant genetic variation.

### Experimental conditions had the greatest influence on differences among leaf spectra

Differences among individual leaves and plants were the largest source of variance, and these were strongly influenced by experimental conditions. The Field_UT dataset showed the largest range of variance, both in the PCA across the whole dataset (Fig. 3a, b) as well as in spectrally resolved coefficients of variation, which were about two times as large (reaching around 40%) across the spectrum (Fig. 5a–c) as for the other two experiments (reaching around 20%). Although the Field_AZ and Glasshouse experiments showed a similar range of variation, CV’s of VIS and NIR spectra were about two times as large in Field_AZ as in the Glasshouse. In the Glasshouse, the TLs group showed a larger CV than the reference group in the SWIR and some water absorption bands. In contrast, differences between TLs and the reference in Field_UT were indistinct and not statistically significant, with the exception of one TL, irCHAL, differing significantly in a narrow wavelength range at the beginning of the VIS (Fig. 7). This supports other studies indicating that leaf spectra can serve as sensitive indicators of plant responses to changing environments, and suggests that control of experimental conditions is important when using spectroscopy to detect subtler genetic differences [2, 7].

The relatively large variation in the Field_UT dataset may result from changes in single leaves over the multiple measurement days, as this was the only experiment in which the same leaves were measured repeatedly. Also, measurements in Field_UT were conducted on-plant outdoors while those for the Glasshouse and Field_AZ were conducted indoors, and so changing environmental conditions during measurements may have contributed to greater overall variance. Consistently with this, measurement uncertainty was about twice as great in Field_UT as in the other two datasets (Fig. 5k), although measurement uncertainty accounted for at most 1% of total variance in each of the experiments.

In the Glasshouse dataset, which had the smallest range of variance (Fig. 3) and the most standardized environment, plants generally looked most similar to each other (Additional file 1: Fig. S1d). Differences were observed in the Glasshouse between the EV reference and TLs, especially the irAOC line, which were not detected for the same lines in Field_UT, and the TLs as a group were more variable than EV plants in the Glasshouse but not in the field (Fig. 7). The plants in the glasshouse were also measured within the smallest time window (Fig. 2b), likely minimizing between-individual variation within each genotype, which had a much larger effect on variance than did measurement time *per se* in our experiments (Fig. 8). One line, irCHAL, differed significantly from the reference in Field_UT, but only for a small range at the beginning of the VIS (400–420 nm) and of our range of measurement, which is subject to relatively higher measurement uncertainty (see Fig. 5d). However, the CHAL gene affects the synthesis of compounds that absorb UV light, and so the difference in this wavelength range near the transition from UV to VIS light is consistent with CHAL gene function [25].

We observed significant effects of measurement time on leaf reflectance in both the Field_UT and Field_AZ experiments (Fig. 8). In the Field_UT experiment, where the same plants were measured at three different time points across three days, we found significant time effects across multiple wavelength ranges, including the VIS, from the red edge to the NIR, and SWIR1 (Fig. 8a). Interestingly, the most significant differences were observed between noon and pm measurements (Fig. 8b). These two time frames may best capture the difference between plants at peak photosynthetic activity, and entering the non-photosynthetic evening period. However, measurements in Field_UT were conducted at a time in the season when the ambient weather conditions ranged from rain to sun, and variation in weather conditions and changes in plants over time contribute to the effect of time in this dataset. In the Field_AZ experiment, where measurements of different plants (no repeat measurements) were conducted over seven consecutive days from dawn to dusk and later put into the same three groups (am, noon, pm) as used in Field_UT, we also observed significant time effects across similar spectral regions (Fig. 8c). However, when we constructed a model using only UT-WT samples, these time effects were no longer significant (Fig. 8d). This suggests that the time effects in Field_AZ may be increased by genetic variation among the PLs and RILs – perhaps including genotypic differences in phenology and environmental responses across the week of measurement – which was not present among the UT-WT samples. Similarly, we found a strong overall batch effect in the Field_AZ experiment, which was significant across almost all wavelengths even after p-value adjustment (Additional file 1: Fig. S5c), but was not observed in the model using only the UT-WT samples (Additional file 1: Fig. S5d), further highlighting the influence of genetic variation on leaf reflectance measurements. Overall, the Field_AZ dataset showed intermediate variance (Fig. 3a, b), which likely resulted from the large number of natural and intercrossed genotypes (351; Fig. 6) as well as the gradient of plant growth across the field plot, which was larger than the plots in Field_UT due to the greater number of plants (Additional file 1: Fig. S1). In sum, our data indicate relatively weak time-of-day effects on leaf reflectance measurements during the photophase (when using a standardized light source and background), and relatively stronger day-to-day and plant-to-plant variation, especially when including multiple genotypes.

A PCA was conducted only on the UT-WT samples from each experiment in order to remove the effect of genotype and thus reveal non-genetic effects. This analysis showed the same relative differences in total variance among experiments, which was again greatest within samples from Field_UT, followed by Field_AZ and the Glasshouse (Fig. 3d, e). This is consistent with the inference that environmental factors were the main determinants of total variance in each dataset. Contributions to PC1 were evenly distributed among wavelengths (Fig. 3f). Thus PC1 may have been mostly influenced by the brightness of the measured signal rather than particular molecular absorption or structural features. Contributions from NIR dominated PCs 2 and 3 while VIS wavelengths dominated PC4. The SWIR1 region, influenced by water absorption and non-pigment dry matter content, contributed more to PCs 2 and 3, and the SWIR2 region contributed more to PC1 and PC3. Spectra from UT-WT plants across the three experiments furthermore differed significantly at most wavelengths, except some water absorption wavelengths following NIR; the SWIR1 region; and a small part of the water absorption bands following SWIR1; with WT samples in the Field_UT experiment differing most from the other two experiments (Fig. 4).

UT-WT samples from the two field environments (Field_AZ and Field_UT) differed from each other primarily in the red edge between the VIS and NIR regions, the NIR, some water absorption bands, and most of SWIR2 (Fig. 4). This likely reflects the difference in phenology and growth form of the plants measured in Field_UT (younger and less bushy) and Field_AZ (older and branchier, Additional file 1: Fig. S1), although we measured leaves at a comparable developmental stage across environments. Mature, non-senescent, light-exposed leaves in Field_AZ, however, came from side branches and were smaller than leaves of the same developmental stage in the other two environments, and so sets of two leaves were measured in Field_AZ when necessary to fill the measurement window, which especially affected the NIR and many water absorption bands between NIR and SWIR1 (Fig. 9a). Nevertheless, Field_UT measurements differed more from Glasshouse measurements than did Field_AZ, even though the measurement approach was most similar for UT and the Glasshouse (on-plant) and the structure of the measured plants was also most similar (younger and less branchy). The Field_UT and Glasshouse samples differed across the VIS and most of the NIR, in some water absorption bands, and in the SWIR2 region influenced by non-pigment dry matter content. In comparison, UT-WT plants grown in Field_AZ did not differ from glasshouse-grown plants in the NIR, but otherwise differed in the same regions as those grown in Field_UT (VIS to red edge, SWIR2, and water absorption bands). Interestingly, the Glasshouse and Field_AZ samples clustered separately along PC2, both for the entire dataset (Fig. 3a) and for the UT-WT samples only (Fig. 3d); the NIR region of highest reflectance, related to leaf structure, contributed more to PCs 2 and 3 than to the other PCs.

Finally, we analyzed the effect of leaf position on reflectance in a small side experiment in Field_UT (Fig. 9c, d), using the reference UT-WT. This analysis indicates that different leaf positions, which in this experiment are correlated with leaf age and not correlated to sun exposure, did not differ significantly when comparing younger, intermediate, or older leaves after p-value adjustment. Position mostly affected the VIS region into the red edge (514–714 nm), which tended to differ between younger versus older leaves. Within each experiment described in this study, leaves at a comparable physical position, light exposure, and developmental stage were used in order to avoid such effects.

In summary, non-genetic factors had the largest influence on variation among measured leaf spectra, and these effects were spread across the spectrum. Thus, caution is advised when comparing leaf spectra datasets from different experiments, and it is challenging to identify signatures of genetic variation in spectra across environments and experiments: genetic variation may not be a dominant feature, but a subtler facet of the dataset. Across all experiments, genetic variation seemed to most influence variation in parts of the VIS and SWIR regions and the red edge between VIS and NIR, and less consistently in the NIR.

### Specific aspects distinguish leaf spectra of different genotype groups

Together, the Field_AZ and Glasshouse data indicate consistently greater variation in the VIS range among the PLs and derived RILs, in comparison to the UT-WT and derived EV reference genotypes, and the other TLs derived from the UT-WT (Figs. 5a–c and 6). It is not surprising to find, on average, more visible variation among natural accessions and their derived offspring, than among inbred plants and otherwise-isogenic TLs targeting genes not directly involved in pigment or wax production. This is often one reason to prefer the use of genetically modified, otherwise isogenic plants to elucidate ecological gene function [62]. More specifically, PLs and RILs in the Field_AZ experiment varied more than the UT-WT reference in the VIS range between 400–537 nm and 662–700 nm, and PLs showed more variability compared to UT-WT reference across the whole VIS in the Glasshouse. This corresponds to violet, blue, blue-green, and red light, and is within the overlapping region of absorption for chlorophylls a (absorption maximum at ca. 440 nm) and b (absorption maximum at ca. 470 nm), together with carotenoids (spectral feature at 520 nm) [63]. These results contrast with a recent phylogenetic analysis of plant spectra indicating that evolution is most tightly constrained in this part of the visible spectrum, and rapidly progresses towards optimization [13]. This contrast may be resolved by the difference in focus of our dataset, which analyzes variation across 360 genotypes of one species replicated across environments, as compared to macro-evolutionary studies focused on species means. Intraspecific variation and micro-evolutionary processes cannot be inferred from data on macro-evolutionary patterns [64–66]. Our results characterize distinct natural variation in the optical properties of *N. attenuata*, which may reflect underlying differences in various leaf characteristics, potentially including the accumulation of pigments such as chlorophylls and carotenoids.

In the Glasshouse, the red edge and much of the NIR region, from 694 nm to 1142 nm, also significantly differentiated the PLs from the reference genotype. The RIL population generated from the PLs, which was grown only in Field_AZ, differed significantly from the reference genotype across the VIS, NIR, and water absorption bands right before SWIR1 (413–1528 nm). Although the RILs differed significantly from the reference across a much greater range of the spectrum than did the PLs, the RILs never differed significantly from the PLs from which they were derived (same genetic material). Generally, greater variation in spectra across specific regions indicated greater genetic variability within both the Field_AZ and Glasshouse experiments (Fig. 6c, d, f), which was best detected in the VIS (dominated by differences in pigments) and around the transition from NIR to SWIR (explained by variation in leaf structure, water absorption, and non-pigment dry matter content). The much larger region of significant difference for the RILs vs. the reference, as compared to their parental lines vs. the reference, may simply be due to the much larger number of RILs sampled, as indicated by the comparison of bootstrapped CVs in Fig. 6.

In contrast, the TLs, which were genetically identical to the reference genotypes except for the insertion of a specific small construct designed to knock down expression of a target gene and thereby manipulate specific traits, mostly did not show any significant differences to the reference (EV) either in the Glasshouse, or in the field (Field_UT). In Field_UT, irCHAL differed significantly from the EV reference in the main experiment across a small window of 20 nm at the beginning of the measurement range, and there was no region of the spectrum where the CV of reflectance among TLs was systematically greater than for the reference EV genotype, when controlling for differences in sample number (Fig. 7a, b). By far the greatest region of spectral variation in Field_UT was in the SWIR, peaking between SWIR1 and SWIR2 and at the end of the spectrum following SWIR2.

In the Glasshouse, only one TL, irAOC, differed significantly from the reference genotypes (Fig. 7c), from the longer NIR wavelengths to water absorption bands following SWIR1 (1135–1889 nm), and most of SWIR2 (2034–2384 nm). This line is knocked down in a gene coding for allene oxide cyclase, which catalyzes a rate-limiting step in biosynthesis of the jasmonate plant hormones: key regulators of specialized metabolites which mediate resistance against insects as well as necrotrophic pathogens [14, 41, 67]. Interestingly, the TL differing most from EV in Field_UT (though not significantly) was irLOX3, knocked down in a gene for a lipoxygenase enzyme which is also a rate-limiting step in jasmonate biosynthesis, upstream of AOC [40]. Thus among the eight TLs manipulated in the expression of genes affecting hormone production, phenolic accumulation, leaf structure, and photosynthesis (Table 1), manipulation of the jasmonates had the greatest effect. In the Glasshouse, the TLs as a group tended to be more variable than the reference in the same spectral regions that significantly differed for irAOC (Fig. 7d). These do not overlap with the parts of the spectrum that differed significantly between the PLs and the reference lines in the Glasshouse. Thus in this experiment, the traits manipulated in the selected TLs had a signature different from the natural variation measured among the PLs, even though the PLs were selected to include natural variation in jasmonate signaling [14]. Taken together, this indicates that we were best able to detect natural variation affecting the VIS and NIR parts of the spectrum.

In summary, differences among groups of natural variants (PLs, RILs) were most pronounced in the VIS, especially around chlorophyll and carotenoid absorption features (Fig. 6) which are predicted to evolve rapidly towards optima across plant species [13]. The transgenic lines, selected for their differences in photosynthetic rates but also protein content as well as contents of various non-pigment constituents (Table 1), indeed showed greater variation in SWIR wavelengths related to water absorption and non-pigment dry matter. Only the irAOC line deficient in jasmonate defense hormones and, in a narrow part of the blue range, the irCHAL line deficient in phenolic acccumulation, showed significant differences compared to reference genotypes. Variation distinguishing the TLs was masked by between-plant differences in the Field_UT experiment, where overall variation was greatest (Fig. 7).

### Limitations and outlook

We analyzed measurements of leaf reflectance from three different experiments including 360 characterized, homozygous genotypes of one wild plant species, *Nicotiana attenuata*. These experiments were conducted at two different field locations and in a third, glasshouse location, and plants were accordingly grown and measured at different times of year. Although the same measurement instruments and procedures for determining leaf reflectance were used, aspects of the measurement approach also varied according to the experimental size and set-up.

We were not able to test directly for effects of all variables that may have affected leaf reflectance in our dataset. We did not compare measurements conducted on-plant directly with measurements of hydrated, cut leaves, an approach which varied by experiment, as did the placement of the instrument in a more controlled indoor environment versus carrying it from plant to plant outdoors. Differences were observed in water absorption bands across all three experiments regardless of whether leaves were measured on-plant, or cut and hydrated; and on-plant glasshouse measurements were more similar to cut-leaf measurements from Field_AZ than to on-plant measurements from Field_UT (Fig. 4b). Measurement uncertainty, which is affected by instrument stability and thus indoor versus outdoor measurement environment, never exceeded 1% of total variation in any experiment (Fig. 5d).

The“effects of time” in our study were under specific conditions: in Field_UT, we measured the same set of plants in morning, noon, afternoon but on different days; in Field_AZ, measurements were conducted over seven consecutive days from dawn to dusk, without repeating any measurements on the same plants. The time effect in Field_AZ therefore incorporated not only different days, but also a randomized distribution of different genotypes. To address this, we constructed an additional model in Field_AZ using only the UT-WT samples. Limiting time, as well as measurement batch analyses to include only the reference genotype eliminated all significant effects of both factors. Leaf position, a proxy for developmental stage rather than light exposure in this study, likewise had a relatively small and non-significant effect on leaf spectra within the reference UT-WT genotype (Fig. 9). Plants in both the Glasshouse and Field_UT had a simple growth form with one main stem allowing to select single large leaves at comparable developmental and stem positions across plants, whereas the bushier growth form of plants in Field_AZ led us to choose leaves on side stems of comparable height and mature, non-senescent developmental stage, but which varied more in size (also within the reference UT-WT genotype). As a result, we sometimes used two leaves for measurements in Field_AZ. When comparing across all reference, PL, and RIL genotypes in the Field_AZ experiment, this had significant effects on part of the green-red window of the VIS (508–641 nm, 693–707 nm) which is the local reflectance maximum; the local reflectance maxima of the red edge and NIR region (731–1149 nm), the water absorption band between the NIR and SWIR1 (1400–1539 nm), and the SWIR2 from 2344 nm till the end (Fig. 9a). Although effects of leaf number were not significant when including only UT-WT samples in Field_AZ, unadjusted p-values showed a consistent pattern with the results of the model including all genotypes. Because this includes several of the regions varying most among genotype groups, it is important to consider accounting for differences in leaf number (one or two) used to fill the measurement window when analyzing specific genotype-phenotype associations in this dataset.

In this study, we have not yet investigated specific genotype-phenotype associations (e.g. by GWAS or QTL mapping), but first assessed overall effects by genotype group versus other influences. Specific genotype associations with candidate genes and loci will in some cases be stronger than the overall, subtle effects presented here; these stronger effects can be reliably assessed if important confounding factors are taken into account, and specific associations can be verified by gene functional studies. Here, our main aim is to assess the spectral signatures and relative influence of these confounding factors, versus spectral variation which seems to be more consistently associated with genetic variation. The bands from ca. 400-530 nm (violet to blue light) and from ca. 660-700 nm (red light), which showed consistent differences between RILs and PLs versus the reference genotype, may give the most reliable indication of genetic variation in our dataset, by avoiding conflation with other experimental effects.

Removing outliers from our analysis, using the traceable procedure described and justified in the methods section, did significantly influence our results. For instance, in the Field$\cdot$UT dataset, two of the five plants removed during the final visual inspection (step 3) were UT-WT samples. The inclusion of these outliers notably altered the shape of the CV curve, particularly in the SWIR2 region, leading to a pattern distinct from other genotypic groups. Upon their removal, the CV for the UT-WT group aligned more closely with the patterns observed in other groups, suggesting these two samples were likely anomalies rather than representative data points. We acknowledge that outlier removal can be subjective and may impact the analysis results. We thus detailed our outlier detection and removal process in the Methods section and an associated Additional file 1 figure for transparency and replicability. Careful identification and removal of outliers are crucial for accurate data interpretation, as their inclusion can distort results.

While our study focused on individual leaf reflectance measurements using a contact probe, it is important to acknowledge the role of leaf angle in remote measurements of canopy reflectance. Leaf angle can significantly affect the distribution of light within the canopy and consequently, the reflectance measurements obtained from remote sensing platforms [68, 69]. This is particularly relevant when scaling up from leaf to canopy or landscape level measurements, where the complexity of the canopy structure, including leaf angle distribution, can introduce additional variability [70]. However, in our study, the influence of leaf angle was minimal as we conducted direct measurements of individual leaves using a standard measurement orientation. Future studies aiming to link leaf-level measurements with canopy or landscape-level reflectance should consider the role of leaf angle and other structural characteristics of plant canopies, which are also subject to both genetic and environmental influences.

## Conclusion

Imaging spectroscopy can support more frequent and widespread biodiversity monitoring, including assessment of the spatial genetic diversity which encodes the current potential of species populations to adapt to global change. This study dissected variation due to common experimental factors using a large and controlled set of plant genotypes under field and glasshouse conditions. Our dataset shows the dominating effect of experimental and environmental factors on within-species differences in leaf reflectance spectra across most wavelengths, except for parts of the SWIR1 and some water absorption bands. However, differences related to natural genetic variants were most pronounced in parts of the VIS and NIR regions. In particular, pigment absorption regions varied consistently in natural genetic variants compared with a reference genotype across environments, and this variation could be distinguished from experimental effects within environments. In contrast, differences among otherwise-isogenic transgenic lines targeting several genes involved in photosynthesis, development, and environmental responses were clearest in the SWIR region, and only under the most standardized plant growth conditions.

Overall, and despite the large influence of experimental conditions, more genetically variable groups (PLs and RILs; and TLs in some cases) showed greater variation in leaf reflectance when compared with a fully isogenic reference population under the same conditions. The time of measurement (day, time of day) and the number of leaves measured both exhibited significant effects across different wavelength ranges, which varied slightly between models and datasets and were magnified by variation among genotypes, but were not necessarily significant within genotypes. Perhaps surprisingly, the leaf developmental stage had no significant effects in our study, whereas between-individual variation and plot effects were larger, especially when including multiple genotypes, and distributed across the spectrum. Our analysis thus indicates that more genetically variable plant populations also vary more in their leaf spectra under field and glasshouse conditions, and shows the importance of standardization across several experimental variables, randomization and blocking, and including appropriate controls (ideally, using an inbred reference genotype) to support the interpretation of genetic variation from leaf spectra, especially across experiments.

### Supplementary Information

**Additional file 1**. **Figure S1.** Example layout of measured plants in **a** Field AZ, **b** Field UT, **c** Glasshouse. Different colors indicatedifferent lines as described. **d** Photos of each environment. The inset of the middle figure shows a UT-WT plant inField UT. Photos by Meredith C. Schuman, pictured: Ewa A. Czyz. **Figure S2.** Examples of outlier detection at each step. **a** Ten scans from the Field AZ white reference (WR) measurementwere identified as outliers in step 1 due to an unexpected leaf-like spectral shape. These two plants were removed from thedataset. **b** In step 2, the LOF method flagged two scans from the Glasshouse black reference measurement as outliers,which were subsequently excluded from reflectance calculations. **c** In the final visual inspection (step 3), five plantswere marked as outliers in Field UT due to deviations in their VIS and NIR values and unusual readings from the waterabsorption band between NIR and SWIR1 to SWIR2. **Figure S3.** Percentage of explained variances of the first eight dimensions of PCA in **a** Field AZ, **b** Field UT, **c**Glasshouse. In all three experiments, the first four dimensions explain more than 95% of total variances. Distributionof samples along (d)-**e**: PC1 and PC2, and **f**: PC3 and PC4, for each dataset. Shaded regions show 95% confidentintervals for each group, i.e. reference samples(UT-WT, EV) versus non-reference samples(PLs, RILs, TLs). **Figure S4.** Comparisons among reference genotypes and full genotypic effects in Field UT, Glasshouse. **a** ANOVAacross reflectance of UT-WT, pRESC2NC (EV1), and pSOL3NC (EV2) samples in Glasshouse. Each colored shade ingrey, orange, or yellow is the range of reflectance of all samples from the corresponding genotype group (UT-WT, EV1,EV2). Each solid colored line is the coefficient of variation (CV) of all samples in the corresponding group. The pinkdotted line is the p-value of ANOVA on all samples in three groups, the pink dashed line is the p-value after adjustment.**b** TLs compared with EV in the mixed model in Field UT. Each dashed colored line indicates the p-value of thecorresponding TL compared with EV in the mixed model, without adjustment for testing multiple wavelengths. The solidcolored lines show the adjusted p-values of irCHAL (green) and irLOX3 (vermilion) versus EV. All other comparisons ofEV with transgenics become 1 across all wavelengths after adjustment. **c** TLs compared with EV in the linear model inGlasshouse. Interpretations of colors and lines are the same as in (**b**). The solid colored lines show the adjusted p-valuesof irAOC (blue) and irRCA (orange) versus EV. **Figure S5.** Post-hoc pairwise comparison of time and batch effects in Field AZ. **a** P-values from post-hoc comparisons inthe linear model (M2) using all samples. Dashed lines represent p-values for comparisons between different times (yellow:am vs noon, blue: am vs pm, orange: noon vs pm), while solid lines represent adjusted p-values. **b** P-values frompost-hoc comparisons in the linear model using only UT-WT samples. Interpretations of colors and lines are the same asin (**a**). **c** The pink dashed line represents the p-values of the batch effect from the linear model (M2) using all samples,with the solid line showing the adjusted p-values. **d** The pink dashed line represents the p-values of the batch effectfrom the linear model (M3) using only UT-WT samples, with the solid line showing the adjusted p-value.

## Data Availability

All plant lines are available from the Max Planck Institute for Chemical Ecology. The spectral measurement data underlying the results presented in this paper are available as a published dataset [71]. All processed spectral data, metadata and code are provided at the GitHub repository: https://github.com/licheng1221/How-leaves-reflect-genetic-variation.
