# Supplementary material for: Evaluating potential of leaf reflectance spectra to monitor plant genetic variation
Source: Plant Methods. 2023 Oct 14;19:108. doi: 10.1186/s13007-023-01089-9 (PMC10576306; doi:10.1186/s13007-023-01089-9)
Supplement: Supplementary file 1 — Additional file 1. Figure S1. Example layout of measured plants in a Field AZ, b Field UT, c Glasshouse. Different colors indicatedifferent lines as described. d Photos of each environment. The inset of the middle figure shows a UT-WT plant inField UT. Photos by Meredith C. Schuman, pictured: Ewa A. Czyz. Figure S2. Examples of outlier detection at each step. a Ten scans from the Field AZ white reference (WR) measurementwere identified as outliers in step 1 due to an unexpected leaf-like spectral shape. These two plants were removed from thedataset. b In step 2, the LOF method flagged two scans from the Glasshouse black reference measurement as outliers,which were subsequently excluded from reflectance calculations. c In the final visual inspection (step 3), five plantswere marked as outliers in Field UT due to deviations in their VIS and NIR values and unusual readings from the waterabsorption band between NIR and SWIR1 to SWIR2. Figure S3. Percentage of explained variances of the first eight dimensions of PCA in a Field AZ, b Field UT, cGlasshouse. In all three experiments, the first four dimensions explain more than 95% of total variances. Distributionof samples along (d)-e: PC1 and PC2, and f: PC3 and PC4, for each dataset. Shaded regions show 95% confidentintervals for each group, i.e. reference samples(UT-WT, EV) versus non-reference samples(PLs, RILs, TLs). Figure S4. Comparisons among reference genotypes and full genotypic effects in Field UT, Glasshouse. a ANOVAacross reflectance of UT-WT, pRESC2NC (EV1), and pSOL3NC (EV2) samples in Glasshouse. Each colored shade ingrey, orange, or yellow is the range of reflectance of all samples from the corresponding genotype group (UT-WT, EV1,EV2). Each solid colored line is the coefficient of variation (CV) of all samples in the corresponding group. The pinkdotted line is the p-value of ANOVA on all samples in three groups, the pink dashed line is the p-value after adjustment.b TLs compared with EV in the mixed model [file 13007_2023_1089_MOESM1_ESM.pdf]

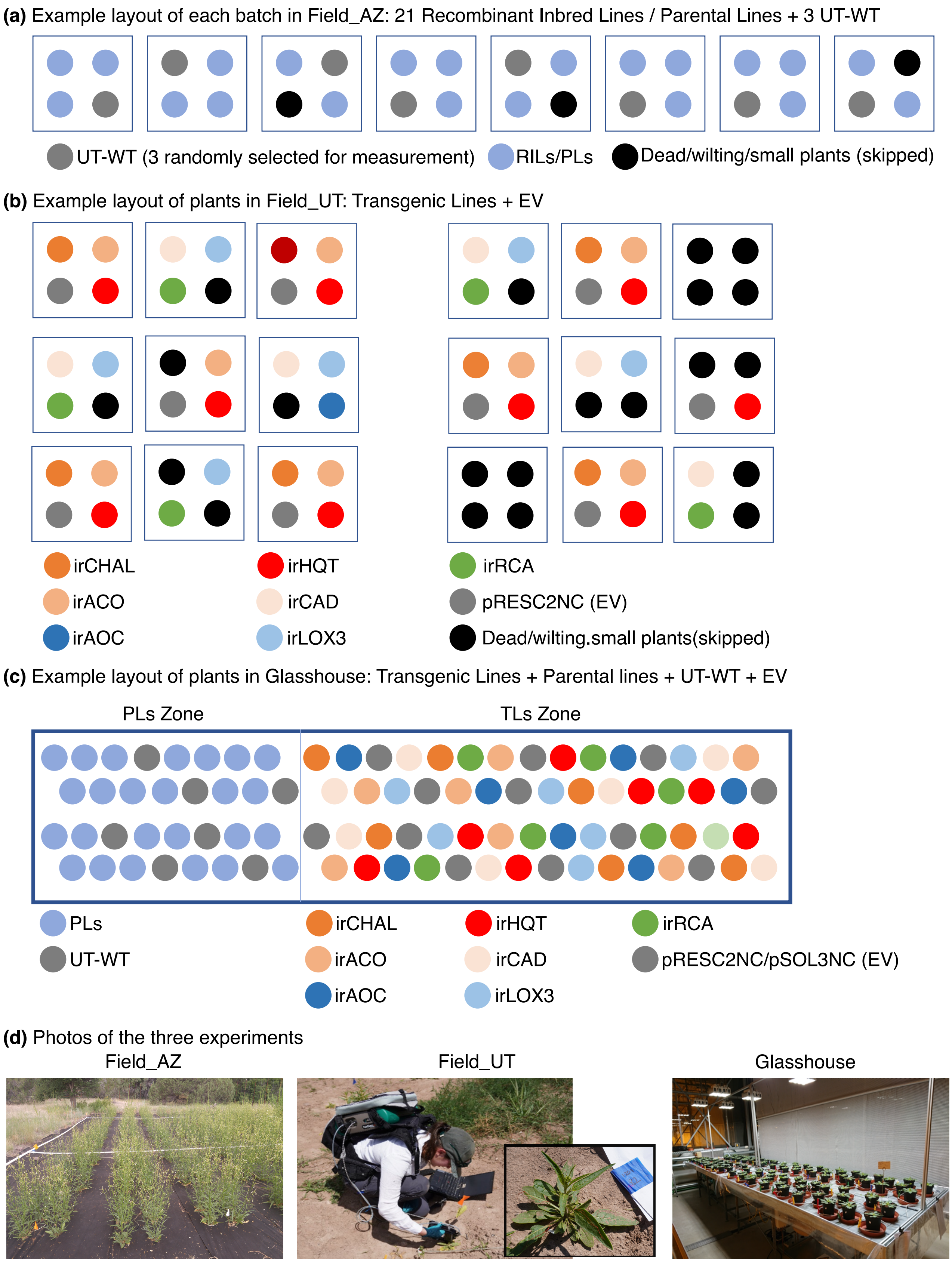

Figure S1: Example layout of measured plants in (a) Field_AZ, (b) Field_UT, (c) Glasshouse. Different colors indicate different lines as described. (d) Photos of each environment. The inset of the middle figure shows a UT-WT plant in Field_UT. Photos by Meredith C. Schuman, pictured: Ewa A. Czyz.

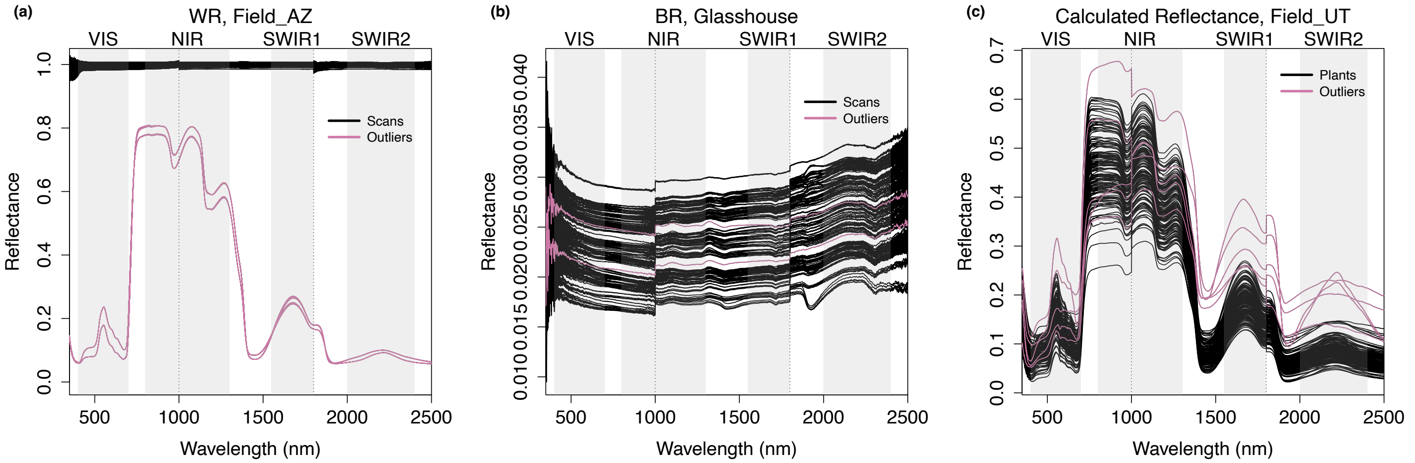

Figure S2: Examples of outlier detection at each step. (a) Ten scans from the Field_AZ white reference (WR) measurement were identified as outliers in step 1 due to an unexpected leaf-like spectral shape. These two plants were removed from the dataset. (b) In step 2, the LOF method flagged two scans from the Glasshouse black reference measurement as outliers, which were subsequently excluded from reflectance calculations. (c) In the final visual inspection (step 3), five plants were marked as outliers in Field_UT due to deviations in their VIS and NIR values and unusual readings from the water absorption band between NIR and SWIR1 to SWIR2.

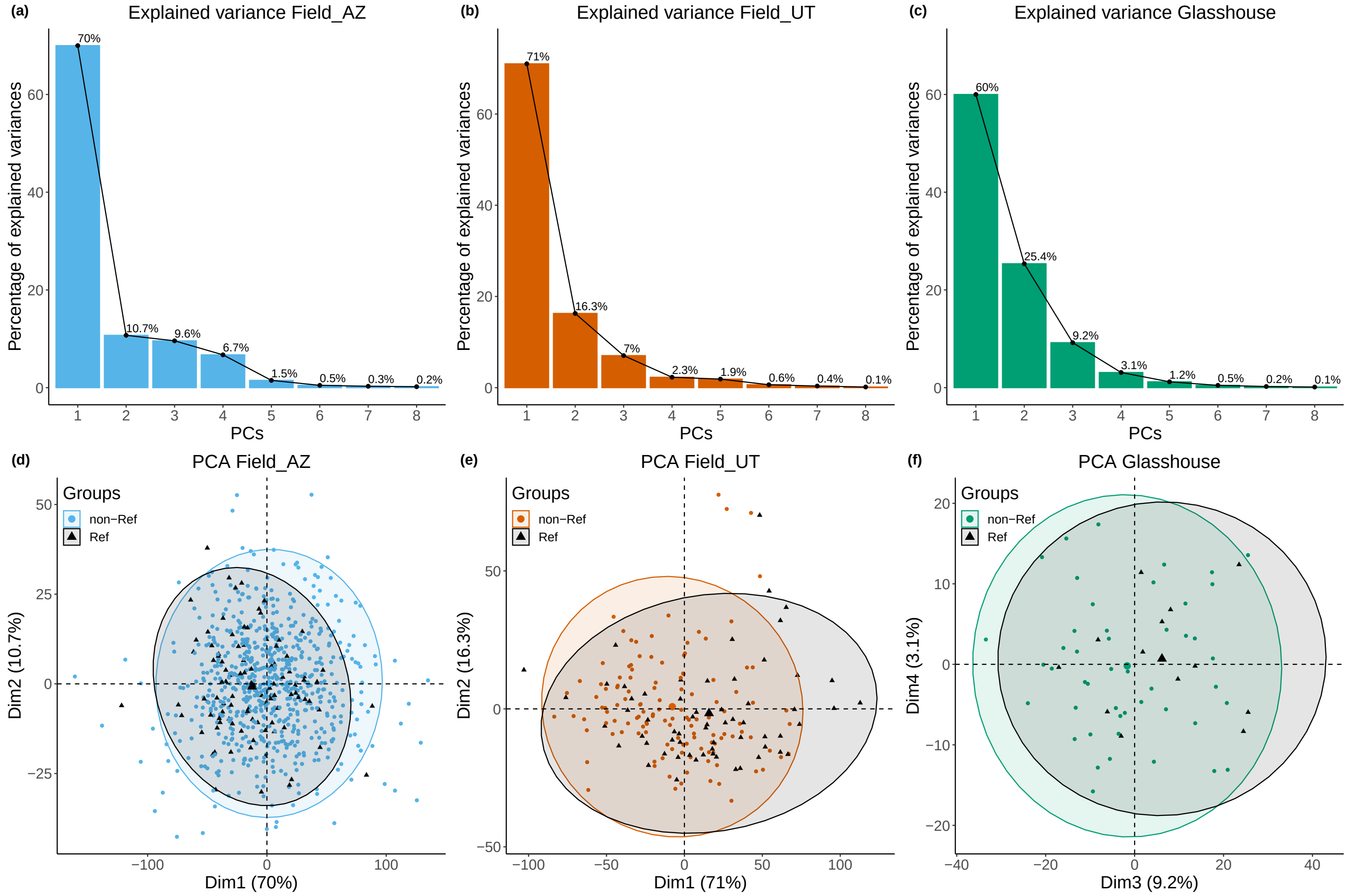

Figure S3: Percentage of explained variances of the first eight dimensions of PCA in (a) Field_AZ, (b) Field_UT, (c) Glasshouse. In all three experiments, the first four dimensions explain more than 95% of total variances. Distribution of samples along (d)-(e): PC1 and PC2, and (f): PC3 and PC4, for each dataset. Shaded regions show 95% confident intervals for each group, i.e. reference samples(UT-WT, EV) versus non-reference samples(PLs, RILs, TLs)

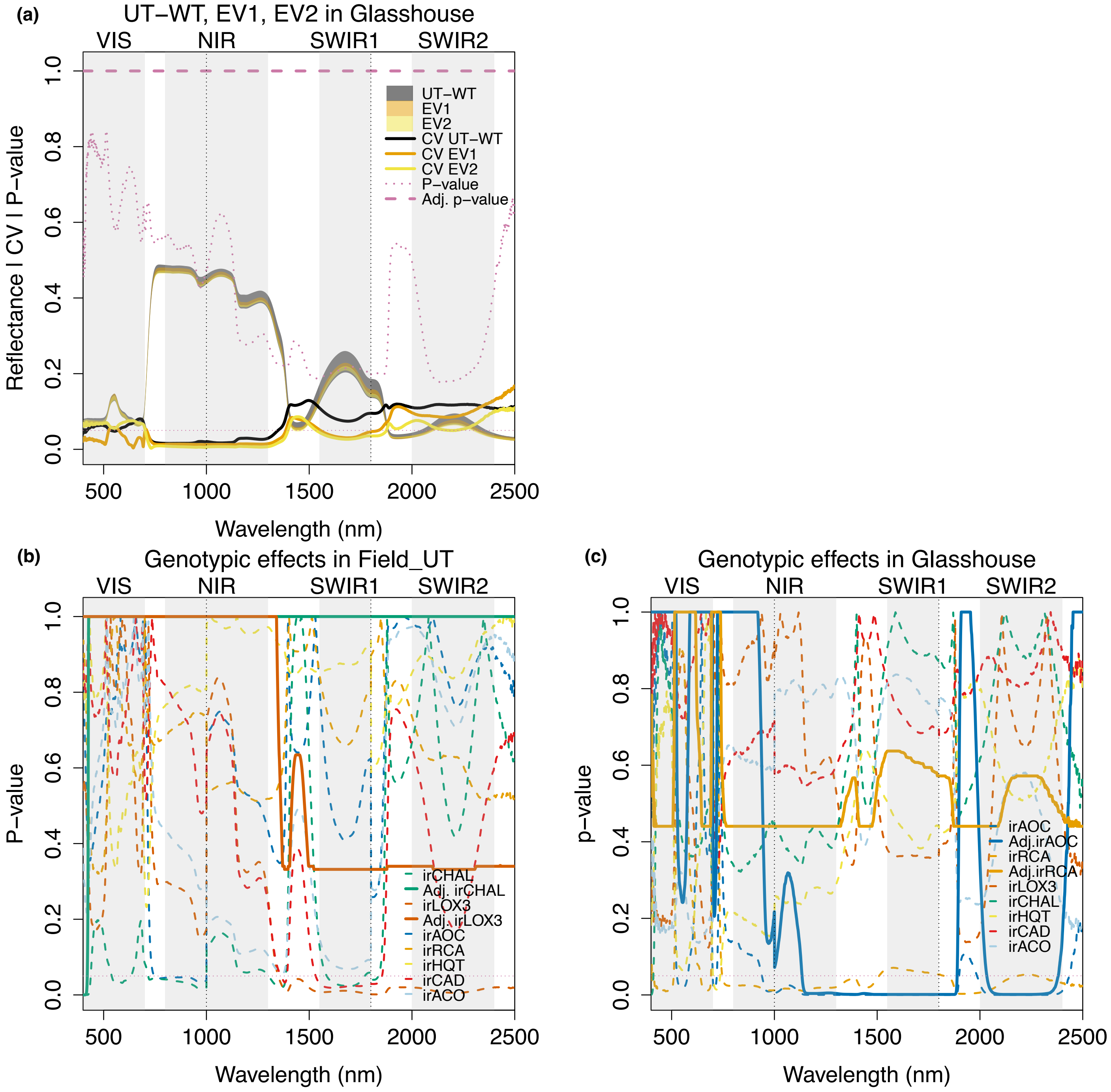

Figure S4: Comparisons among reference genotypes and full genotypic effects in Field_UT, Glasshouse. (a) ANOVA across reflectance of UT-WT, pRESC2NC (EV1), and pSOL3NC (EV2) samples in Glasshouse. Each colored shade in grey, orange, or yellow is the range of reflectance of all samples from the corresponding genotype group (UT-WT, EV1, EV2). Each solid colored line is the coefficient of variation (CV) of all samples in the corresponding group. The pink dotted line is the p-value of ANOVA on all samples in three groups, the pink dashed line is the p-value after adjustment. (b) TLs compared with EV in the mixed model in Field_UT. Each dashed colored line indicates the p-value of the corresponding TL compared with EV in the mixed model, without adjustment for testing multiple wavelengths. The solid colored lines show the adjusted p-values of irCHAL (green) and irLOX3 (vermilion) versus EV. All other comparisons of EV with transgenics become 1 across all wavelengths after adjustment. (c) TLs compared with EV in the linear model in Glasshouse. Interpretations of colors and lines are the same as in (b). The solid colored lines show the adjusted p-values of irAOC (blue) and irRCA (orange) versus EV.

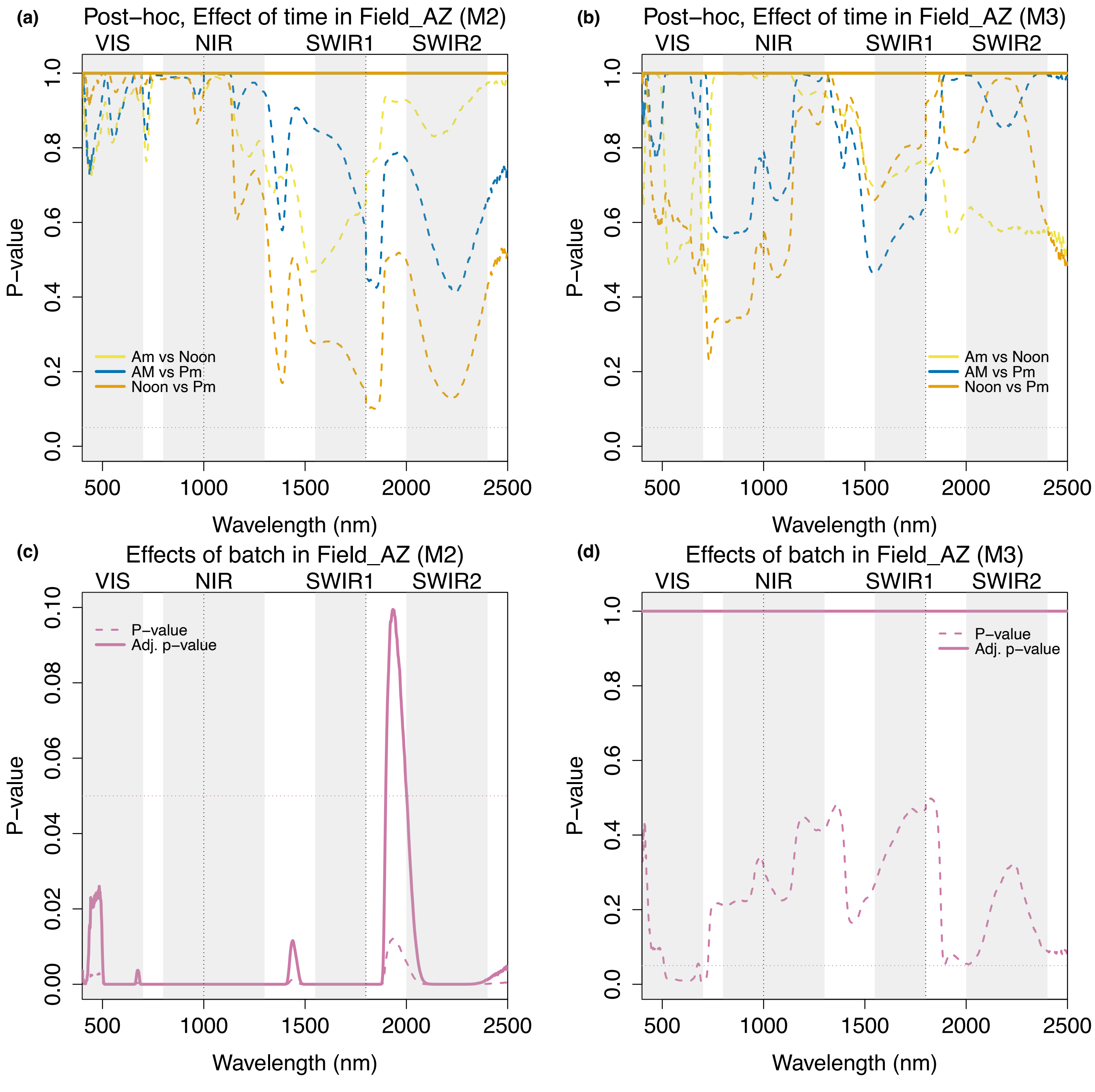

Figure S5: Post-hoc pairwise comparison of time and batch effects in Field_AZ. (a) P-values from post-hoc comparisons in the linear model (M2) using all samples. Dashed lines represent p-values for comparisons between different times (yellow: am vs noon, blue: am vs pm, orange: noon vs pm), while solid lines represent adjusted p-values. (b) P-values from post-hoc comparisons in the linear model using only UT-WT samples. Interpretations of colors and lines are the same as in (a). (c) The pink dashed line represents the p-values of the batch effect from the linear model (M2) using all samples, with the solid line showing the adjusted p-values. (d) The pink dashed line represents the p-values of the batch effect from the linear model (M3) using only UT-WT samples, with the solid line showing the adjusted p-value.
